# Chemically Assisted Precompression of Hydrogen Molecules in Alkaline-Earth Tetrahydrides

**DOI:** 10.1021/acs.jpclett.2c02157

**Published:** 2022-09-02

**Authors:** Miriam Peña-Alvarez, Jack Binns, Miriam Marqués, Mikhail A. Kuzovnikov, Philip Dalladay-Simpson, Chris J. Pickard, Graeme J. Ackland, Eugene Gregoryanz, Ross T. Howie

**Affiliations:** † Centre for Science at Extreme Conditions and School of Physics and Astronomy, University of Edinburgh, Edinburgh EH9 3FD, U.K.; ‡ Center for High Pressure Science and Technology Advanced Research, Shanghai 100094, P. R. China; § Department of Materials Science and Metallurgy, University of Cambridge, Cambridge CB3 0FS, U.K.; ∥ Advanced Institute for Materials Research, Tohoku University, Sendai 980-8577, Japan; # Key Laboratory of Materials Physics, Institute of Solid State Physics, Hefei 230031, P. R. China

## Abstract

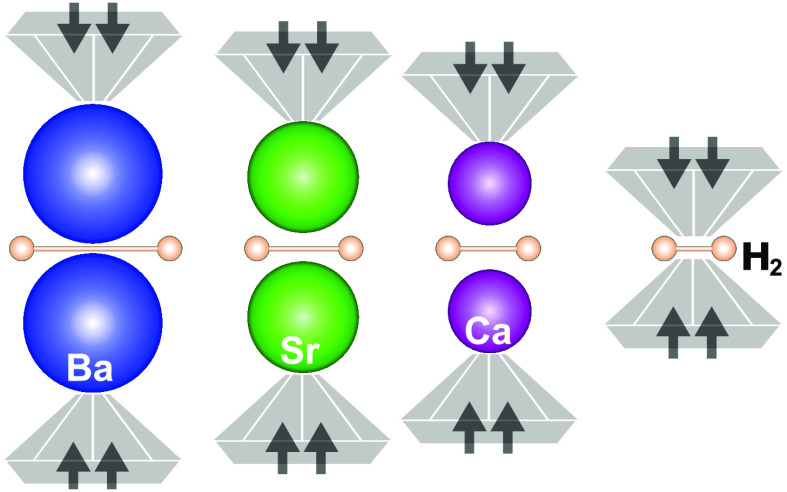

Through a series of high pressure diamond anvil experiments, we report the synthesis of alkaline earth (Ca, Sr, Ba) tetrahydrides, and investigate their properties through Raman spectroscopy, X-ray diffraction, and density functional theory calculations. The tetrahydrides incorporate both atomic and quasi-molecular hydrogen, and we find that the frequency of the intramolecular stretching mode of the  units downshifts from Ca to Sr and to Ba upon compression. The experimental results indicate that the larger the host cation, the longer the  bond. Analysis of the electron localization function (ELF) demonstrates that the lengthening of the H–H bond is caused by the charge transfer from the metal to  and by the steric effect of the metal host on the H–H bond. This effect is most prominent for BaH_4_, where the precompression of  units at 50 GPa results in bond lengths comparable to that of pure H_2_ above 275 GPa.

The application of high pressure provides a mechanical route to break covalent bonds and obtain otherwise unachievable chemical states. Hydrogen is the archetypal example: the 1935 prediction of a molecular–atomic transition at extreme compression has inspired decades of experimental research.^1−6^ Current data suggests that hydrogen still retains its molecular bond up to pressures of at least 360 GPa,^7−9^ pushing the limits of conventional diamond anvil cell experiments. Chemical dopants can assist in the dissociation of quasi-molecular H_2_ units by two mechanisms: electronic effects (charge transfer) and confining the molecule to small interstitial locations, the so-called chemical precompression.^10−12^ This principle has been extensively used in the production of high temperature superconducting hydrides.^13,14^ However, an understanding of the mechanisms involved in H_2_ precompression and how the H_2_ bonding distances are adjusted by chemical doping have yet to be achieved. In the majority of diamond anvil cell studies reporting the synthesis of metal hydrides, usually only the structure of the host metal can be experimentally determined. This is due to the comparatively weaker X-ray scattering of hydrogen and to the fact that the synthesis conditions of many interesting hydride candidates cannot be routinely reached in neutron diffraction studies. As such, often important information, such as the hydrogen content and the interatomic hydrogen distances, is reliant on theoretical calculations.^15−17^ If the hydride contains H_2_ units, then Raman spectroscopy can be an efficient method to investigate the high pressure response of the H–H distances of the molecules. Out of the many known binary metal–hydrogen compounds (group I to XVII), only sodium trihydride and calcium tetrahydride are known to host quasi-molecular hydrogen.^18−20^ In these cases, the Raman frequency of the H–H stretching mode (vibron) exhibits a downshift compared to pure molecular hydrogen, and this is related to a lengthening of the bond.

The alkaline-earth metals, except for radium, react readily with hydrogen, forming binary hydrides, MH_2_ (M = alkaline-earth metal).^21^ CaH_2_, SrH_2_, and BaH_2_ undergo the same structural phase sequence on compression, from the cotunnite structure (*Pnma*, phase I),^22−25^ to the hexagonal Ni_2_In structure (*P*6_3_/*mmc*, phase II). A further transition to an AlB_2_-type structure (P6/*mmm*, phase III), has been experimentally observed in BaH_2_.^21,23,24,26^ The structural transitions of the dihydrides are shifted to lower pressures for the heavier alkaline earth metals; e.g., phase III of CaH_2_ is predicted at 180 GPa, while phase III of BaH_2_ is observed between 40 and 50 GPa.^21−26^

Through a combination of both high temperature and high pressure, interesting compositions were observed in the alkaline-earth-metal–hydrogen system. Superconducting CaH_6_ was produced at pressures greater than 160 GPa.^27,28^ Strontium polyhydrides have been reported above 70 GPa with a range of stoichiometries: SrH_22_, SrH_9_, Sr_8_H_46_, SrH_6_, Sr_3_H_13_, and Sr_2_H_3_.^29^ Barium polyhydrides have also emerged, with Ba_8_H_46_ at 50 GPa^30^ and BaH_10_, BaH_6_, BaH_21–23_, and BaH_12_ all above 90 GPa.^31^

Here, we synthesize CaH_4_, SrH_4_, and BaH_4_ in a series of diamond anvil cell experiments and study the influence that the metal host has on the H_2_ bond. While a combination of both temperature and pressure are required to transform the H_2_ embedded dihydrides into CaH_4_ (50 GPa, 1600 K) and SrH_4_ (40 GPa, 1600 K), BaH_4_ is formed on room temperature compression alone above 40 GPa. Synchrotron X-ray diffraction measurements of all three compounds revealed isostructural phases adopting *I*4/*mmm* symmetry. However, *I*4/*mmm* BaH_4_ appears as a metastable intermediate phase: both temperature and time lead to a transformation to the theoretically stable Ba_8_H_46_, while  BaH_4_ forms upon decompression. All of the tetrahydrides share common Raman signatures indicating the presence of  units within the metallic lattice. There is a more pronounced downshift of the  vibrational Raman frequency with increasing size of the alkaline earth cation, and this can be related to an increase in the H–H intramolecular distance. Electron localization function (ELF) calculations suggest that at a given pressure, the radius of the ELF basin around the  molecule becomes smaller with increasing atomic number of the alkaline earth cation, causing the lengthening of the  intramolecular bond. As a result, the intramolecular hydrogen bond lengths of the alkaline-earth tetrahydrides at relatively low pressure (20–50 GPa), are comparable to those of ultradense elemental hydrogen at 220–275 GPa.

Elemental Ca (99.9%, Alfa Aesar), Sr (99.99%, Sigma-Aldrich), and Ba (99%,Sigma-Aldrich) (or BaH_2_ (99.5%, ABCR)) were loaded into diamond anvil cells in an argon atmosphere glovebox and subsequently gas loaded with research grade hydrogen (99.9995%) at 0.2 GPa (see Supporting Information for detailed methods). After sample loading, X-ray diffraction measurements indicated that dihydrides (MH_2_) were formed, adopting the *Pnma* space group. Upon compression, all dihydrides were observed to undergo a phase transformation to *P*6_3_/*mmc* space group, with the transition pressures and phase sequences in agreement with the existing literature, Figure S1.^22,23,26^ Laser heating *P*6_3_/*mmc* CaH_2_ in an H_2_ atmosphere at 52 GPa to temperatures of 1800 K, yielded the previously reported tetragonal *I*4/*mmm* CaH_4_, see Figure 1a (and Figures S1–S4).^19^ After laser heating SrH_2_ within excess H_2_ at a pressure of 44 GPa to temperatures of 1700 K, we observe a complete transformation to *I*4/*mmm* SrH_4_ upon quench; this phase persisted upon decompression to at least 4 GPa (Figure 1a and Figures S1, S2, S3, and S5). Both the structure, and the experimentally observed volumes as a function of pressure (Figure 1b), are in good agreement with our own (Tables S1–S4) and previous DFT calculations.^12,33^ In a subsequent experimental run, we prepared a sample of Sr embedded in ammonia borane. Ammonia borane can act as a hydrogen source, decomposing at high temperature to produce H_2_ and BN.^34^ After an initial laser heating at 75 GPa, SrH_2_ is formed, and after a subsequent heating, SrH_2_ transforms to another compound adopting an  structure; see Figure S6. We find that the lattice parameters and volumes agree well with those for the recently reported cubic SrH_9_; see Figure S1.^29^ Upon decompression, we find that SrH_9_ does not decompose to at least 30 GPa.

**Figure 1:**
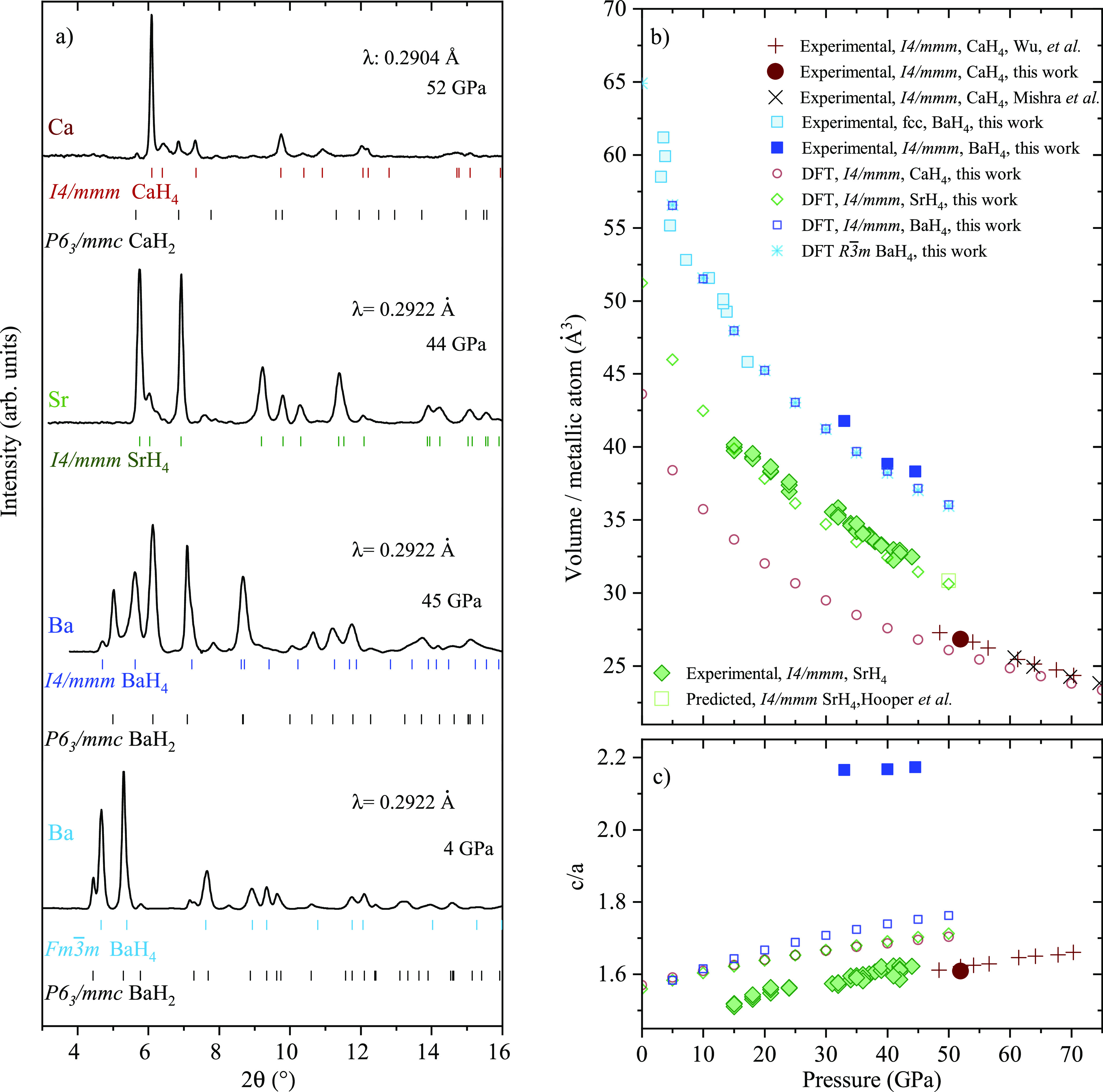
(a) Representative X-ray diffraction patterns of the alkaline-earth tetrahydrides. Tick marks indicate Bragg peaks from the labeled phases. CaH_4_, SrH_4_, and BaH_4_ are refined to tetragonal *I*4/*mmm* at 52, 44, and 45 GPa, respectively. Upon decompression, *I*4/*mmm* BaH_4_ (and Ba_8_H_46_)^30^ undergoes a phase transition to  BaH_4_. (b) Volume per alkaline-earth atom as a function of pressure. Filled symbols correspond to this work, CaH_4_ (brown circles), SrH_4_ (green diamonds), *I*4/*mmm* BaH_4_ (dark blue squares), and  BaH_4_ (light blue squares). Brown crosses and brown asterisks correspond to CaH_4_ from Wu et al.^19^ and Mishra et al.,^20^ respectively. The open green square is the predicted value of SrH_4_ by Hooper et al.^33^ The other open symbols correspond to our DFT calculated volumes. (c) *c*/*a* ratio as a function of pressure for the *I*4/*mmm* structural types.

On room temperature compression of BaH_2_ + H_2_, we observe the formation of BaH_4_*I*4/*mmm* above 40 GPa (Figure 1 and Figures S1–S3 and S5). Although CaH_4_ and SrH_4_ also adopt an *I*4/*mmm* structure, the *c*/*a* ratio of 2.17 for BaH_4_ is considerably larger than that of either SrH_4_ or CaH_4_, where *c*/*a* ≈ 1.6 (see Figure 1c). In all experiments, the transformation to *I*4/*mmm* BaH_4_ is incomplete, and a considerable fraction of BaH_2_ was present. Instead of a complete transformation to *I*4/*mmm* BaH_4_ with time, there is a kinetically sluggish transformation to the documented clathrate type-I  Ba_8_H_46_. If the *I*4/*mmm* BaH_4_ sample is laser heated, then there is a complete transformation to Ba_8_H_46_.^30^ Upon decompression of either *I*4/*mmm* BaH_4_ or  Ba_8_H_46_, a new phase emerges below 27 GPa that can be indexed to an  structure and is stable to at least 4 GPa (see Figures 1 and S3 and S5). The volume per Ba atom as a function of pressure of the  structure is similar to that observed for *I*4/*mmm* BaH_4_, Figure 1b.

Structural searches using AIRSS^35^ result in a number of candidate structures for BaH_4_ with similar energy (all small bandgap insulators, Figures S7–S9). *I*4/*mmm* BaH_4_ is always competitive but never stable, while  is found to be the most stable structure below 25 GPa. The instability of *I*4/*mmm* BaH_4_ in both DFT and experiments suggest that this is an intermediate phase formed as hydrogen migrates through the Ba lattice. The *I*4/*mmm* and  predicted structures are subgroups of  in which the symmetry-breaking is generated by the orientation of the H_2_ molecules. In the absence of detectable hydrogen scattering, the XRD pattern can be equally well indexed to either  or . Starting from the  structure, we observe in *ab initio* molecular dynamics calculations that H_2_ at 300 K went through a number of reorientation transitions on a picosecond time scale. Such reorientations indicate that BaH_4_ will have  symmetry at 300 K, with rotationally disordered  molecules.

To investigate the bonding, we study the ELF.^36^ Free electrons have an ELF value close to 0.5, while higher ELF values indicate that the electrons are localized, e.g., covalent bonds, lone pairs, and atomic shells.^36^ For the empty *I*4/*mmm* Ca/Sr/Ba structures, we identify two ELF maxima types, one centered on the 2*b* sites and elongated along the *c* axis (pseudo-octahedral interstices, Figures 2 and S10) and another type with smaller ELF/volume located on the 4*d* sites, occupying tetrahedral interstices (Figures 2 and S10). The  Ba ELF topology is equivalent to that of *I*4/*mmm* Ba: octahedral ELF maxima with higher ELF values and tetrahedral sites of smaller ELF, Figures 2b and S10. The octahedral maxima of the metallic lattices correspond to the sites hosting the  molecules in the tetrahydrides, and the tetrahedral maxima to the sites hosting the H^–^ anions (Table S5). In -Ba and in *I*4/*mmm*-Ca/Sr the octahedral maxima are almost twice as high as for the tetrahedral ones, showing that these are good sites to localize electrons. However, *I*4/*mmm* Ba has a lower ELF at the octahedral site, with a value almost as low as the tetrahedral site, suggesting that the  units are unfavorable; see Table S5. Indeed experimentally, over time, the *I*4/*mmm* BaH_4_ structure transforms to Ba_8_H_46_,^30^ which has only tetrahedral sites, and to  BaH_4_ in decompression, where the high-ELF octahedral site is more favorable for a molecular hydrogen unit.

**Figure 2:**
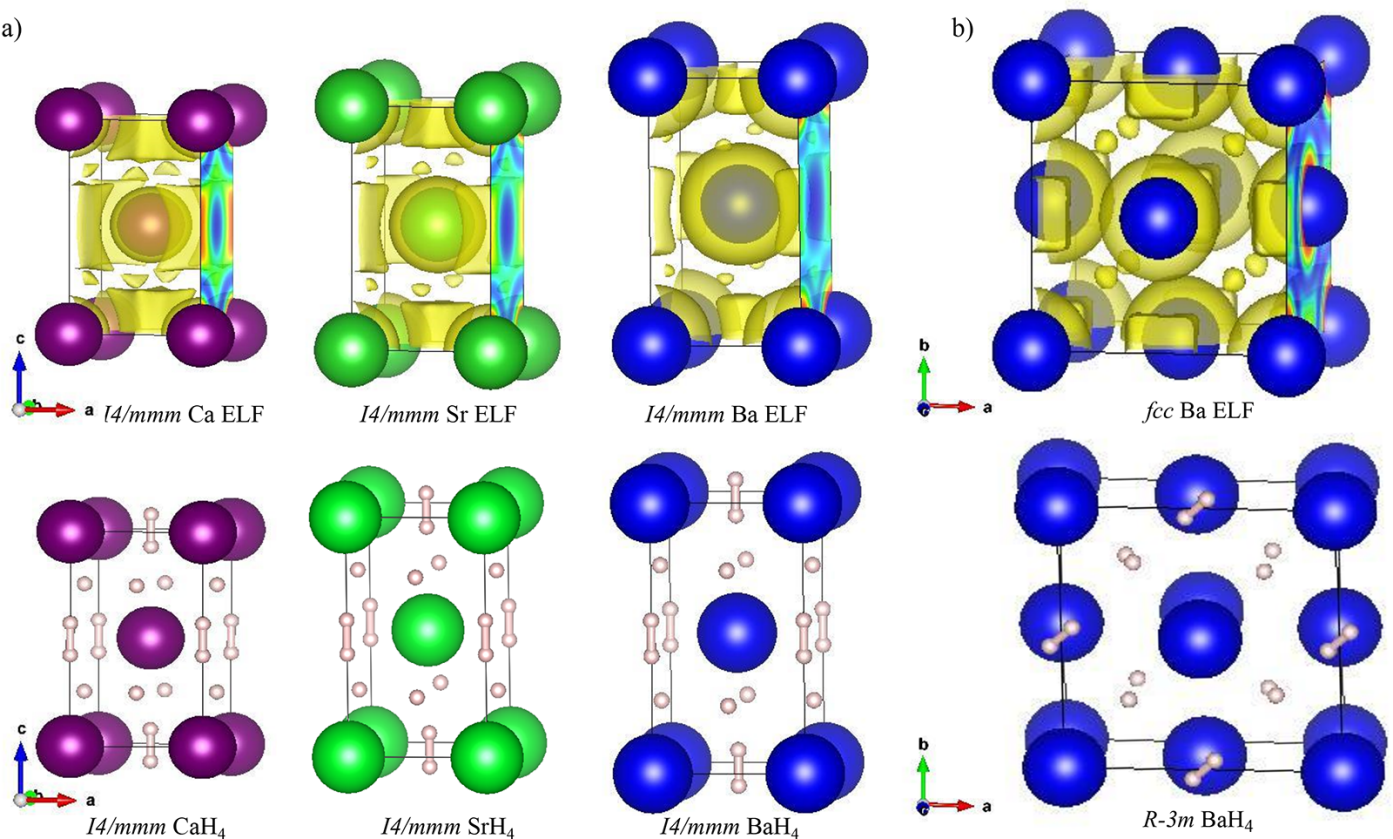
(a) Top: ELF isosurfaces (ELF = 0.44, 0.38, 0.30) for the pure *I*4/*mmm* M sublattices (M = Ca, Sr, Ba) of MH_4_ at 50 GPa (in yellow). Ca, Sr, and Ba atoms are represented as purple, green, and blue spheres, respectively. The ELF values at the octahedral and tetrahedral maxima in Ba (0.477, 0.318) are similar to and lower than those of Ca (0.794, 0.472) and Sr (0.705, 0.406). Bottom: Conventional unit cell of *I*4/*mmm* MH_4_. (b) Top: ELF isosurfaces (ELF = 0.36) (in yellow) of the  Ba sublattice at 10 GPa. The ELF values at the octahedral and tetrahedral maxima are 0.653 and 0.384, respectively. Bottom: crystal structure of  BaH_4_ at 10 GPa. To analyze the experimental , BaH the  space group is used.  BaH_4_ appears as the most stable in calculations at low pressures, and would correspond to  but with orientationally ordered H_2_ molecules.

All of the alkaline-earth tetrahydrides exhibit intense Raman activity, (see Table S6 for DFPT predictions^38^). Experimentally, we see the double-degenerate E_2*g*_ mode between 400 and 1100 cm^–1^), which is attributed to modes associated with the angular degrees of freedom of the , referred as libration.^39,40^ As seen in Figure 4a for all the tetrahydrides, the  libration hardens substantially with pressure. Moreover, there is also a strong dependency of the frequency on the host alkaline earth metal atomic number, with Ba exhibiting the highest upshift. Previous works have calculated the effect on the Raman modes of a single H_2_ molecule when trapped in cages of various sizes.^39^ By this means, compression of the cage leads to an upshift in the libron frequency.^39,40^ This agrees well with the alkaline-earth tetrahydrides hosting H_2_ molecules within octahedral cages, and the libron frequency increases going from larger basin of Ca to the smaller basin of Ba, Figure 2 and Table S5. Both *I*4/*mmm* and  BaH_4_ exhibit similar libron Raman frequencies and pressure dependency, which is understandable as in both configurations the H_2_ molecules are hosted by octahedral cages.

The most intense band, above 3000 cm^–1^ (Figure 3), corresponds to the A_1*g*_ mode resulting from the H–H intramolecular stretching of the , vibron (see Figure 3 and Figure S11).^19,33^ The  vibron frequency and bandwidth depends strongly on the hosting cation. The Raman spectra presented in Figure 3 were measured during decompression of the tetrahydrides (see also Figure S12). At 25 GPa, the intramolecular vibron has a frequency of 3800 cm^–1^ for CaH_4_, 3750 cm^–1^ for SrH_4_, and 3500 cm^–1^ for BaH_4_. The  vibron broadens with pressure, an effect observed in pure H_2_ and attributed to the reduced lifetime of the molecule.^3^Figure 4b shows the Raman shift of the  vibron as a function of pressure together with a comparison with pure H_2_.^3^ In all the alkaline-earth tetrahydrides, the  vibron softens rapidly in compression, more markedly as the alkaline earth atomic number increases.

**Figure 3:**
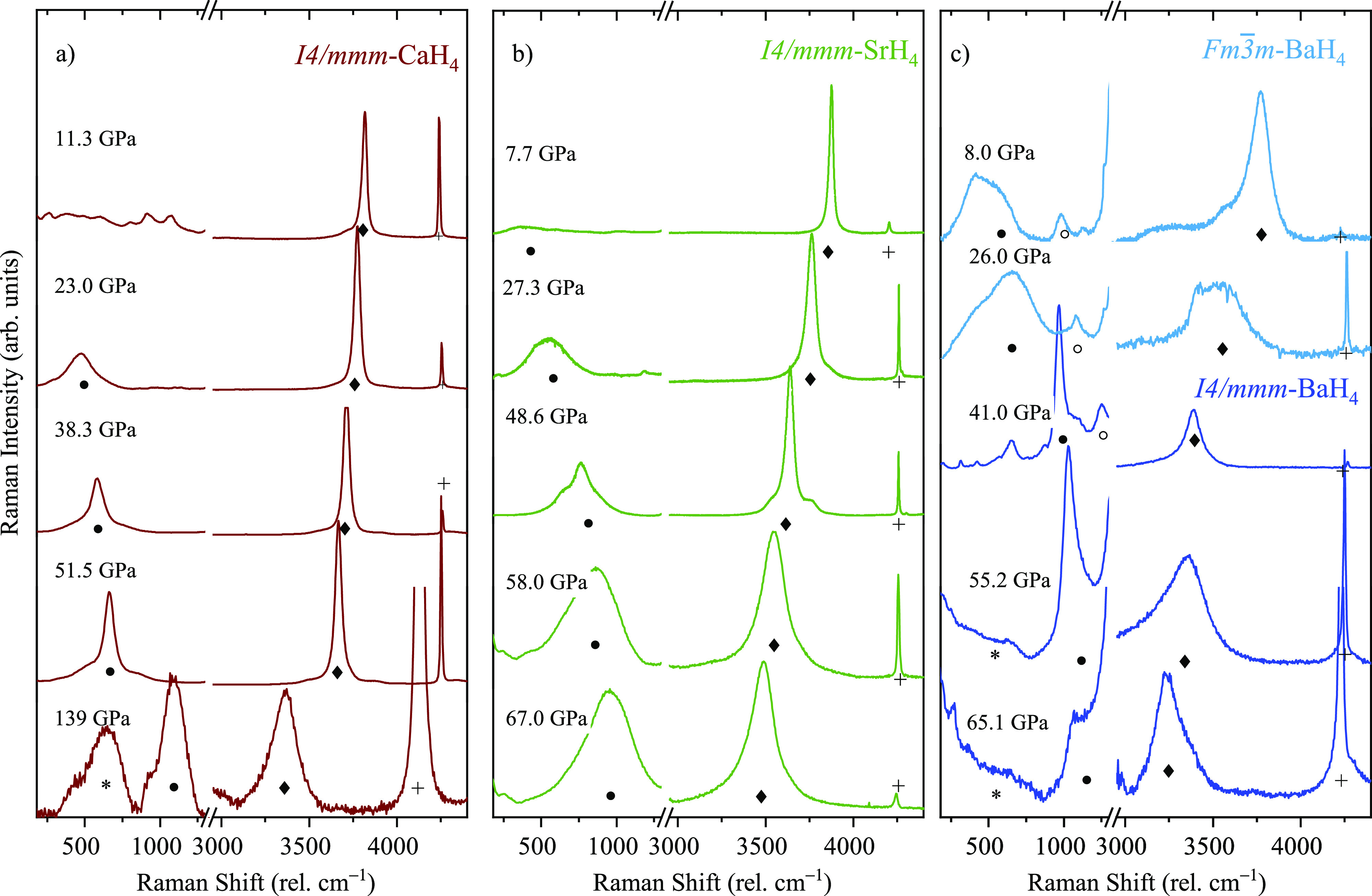
Raman spectra of the alkaline-earth tetrahydrides upon decompression: (a) CaH_4_ + H_2_, (b) SrH_4_ + H_2_, and (c) BaH_4_ + H_2_. The A_1*g*_ vibrons are marked with solid diamonds. Solid circles mark the E_2*g*_ libron, while the empty circle indicates the E_2*g*_ mode corresponding to an H_2_ libron coupled to a H^–^ lattice mode observed only in BaH_4_. Crosses and asterisks mark the rotational modes and vibron of excess pure hydrogen, respectively.^3^

When pure molecular hydrogen is compressed to above 30 GPa, there is a turnover in the intramolecular vibron frequency and a lengthening of the H–H bond.^41−45^ For instance, the H–H bond lengthens from 0.718 at 6 GPa in phase I to 0.84 Å when entering phase IV at 225 GPa,^3,46,47^ and this is manifested by a 650 cm^–1^ drop in the H_2_-ν_1_ frequency.^3^ Comparing the  vibron in the alkaline tetrahydrides with the vibron of pure H_2_-ν_1_, we observe that pressures of only 60 GPa for CaH_4_, 40 GPa for SrH_4_ and 20 GPa for BaH_4_ are sufficient to reach values seen in H_2_ at 225 GPa (Phase IV). Using the empirical formula relating the Raman shift of the H–H vibron with the bond lengths (*r*), *νr*^3^ proportional to a constant, and the H–H bond length of pure H_2_ at 6 GPa as a reference,^37^ we plot the H–H bond lengths as a function of pressure in Figure 4c, together with the bond lengths calculated from our DFT calculations. If the theoretical optimization of the internal coordinates of *I*4/*mmm* BaH_4_ is constrained to the experimental lattice parameters, the H–H distances are larger (e.g., 2.2% at 50 GPa) but still retain the molecular bond. Even at the lowest pressures reached experimentally in this study, all the tetrahydrides show H–H distances corresponding to those of pure H_2_ above 220 GPa. Compared to other metal hydride systems, NaH_3_ exhibits a downshift of the  vibron, at around 4100 cm^–1^ in contrast to 4250 cm^–1^ in pure H_2_ at 50 GPa.^18^ This further demonstrates that the effect of H_2_ chemical precompression is more pronounced in systems with heavier metallic hosts.

**Figure 4:**
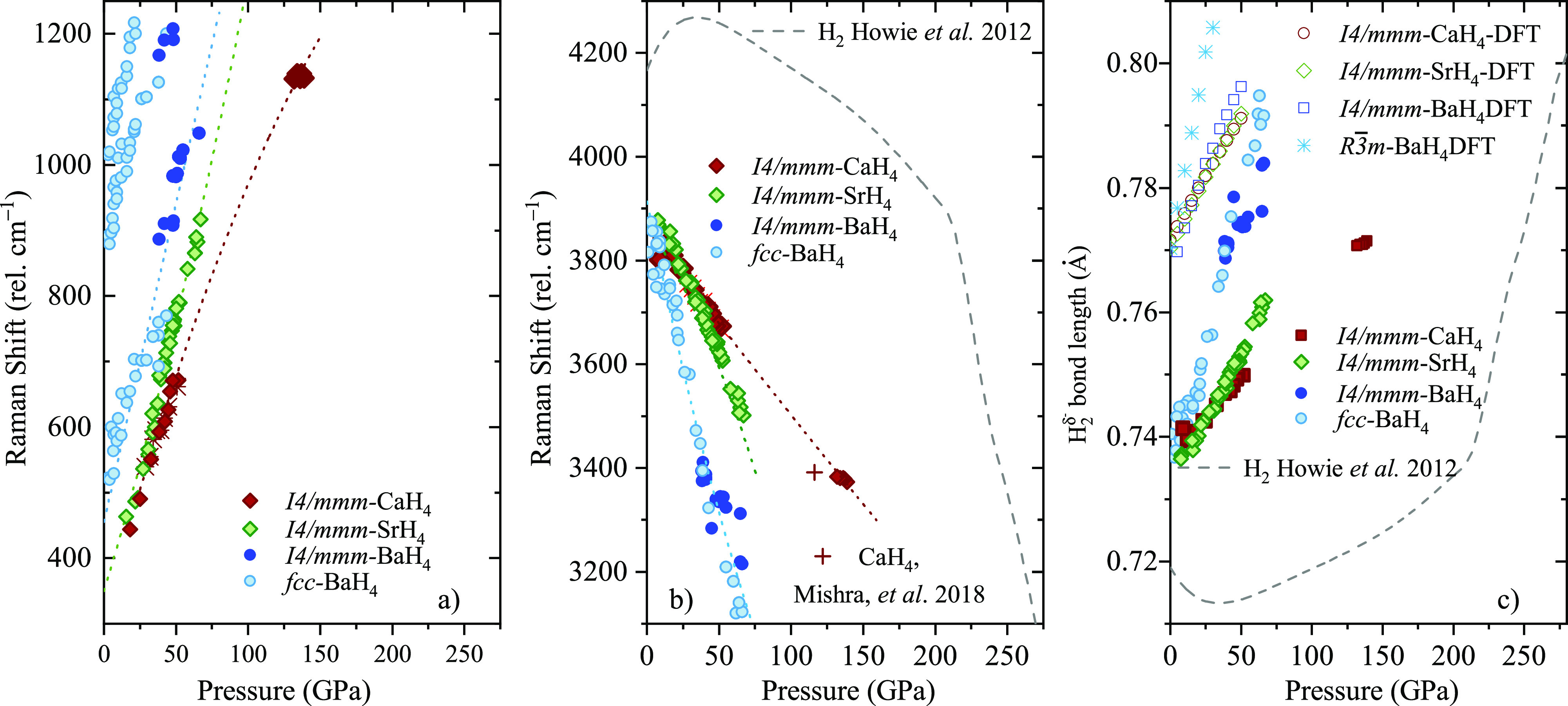
(a) Raman shift as a function of pressure of the E_2*g*_ librational modes. The dotted line is a guide to the eye. (b) Raman shift as a function of pressure of the A_1*g*_ vibron. The dashed line corresponds to pure H_2_,^3^ the dotted lines provide a guide to the eye. (c) Intramolecular H–H bond length estimates as a function of pressure. Symbols represent the  bond lengths (*r*) calculated using the empirical relationship *νr*^3^, where *ν* is the Raman vibrational frequency.^37^ The dashed line represents the corresponding values of pure H_2_.^3^ Hollow symbols and asterisks correspond to H–H distances obtained from DFT calculations.

It has been long suggested that electron transfer from an electropositive metal to  would contribute to the stretching and weakening of the H–H bond via the population of the σ* orbitals.^11,12^ We have calculated the ELF basin charges and the Bader charges using the Quantum Theory of Atoms in Molecules (QTAIM^48^) for the different MH_4_ theoretically optimized structures, see Figure S13–S15. As shown as a function of pressure in Figure 5a the alkaline earth metal donates electrons to H^–^ and . Ca and Sr charges are similar and higher in value than the Ba charge.^49−51^ The charge of the hydrogen anion, , decreases in compression (in absolute numbers—toward neutral charge) regardless of the structure. Within the *I*4/*mmm* tetrahydrides, the  charge is highest (the most negatively charged) in Sr and then decreases for Ca and furthermore for Ba, while  BaH_4_ is smaller than its tetragonal analogue. Both the positive charge of the metallic cations and the negative charge of the  must contribute to the population of the σ*  orbitals, Figure 5a. However, within the *I*4/*mmm* structure, the  of CaH_4_ is more negatively charged than that in BaH_4_. Consequently, the experimentally observed longest H–H bond of BaH_4_ cannot just be explained by the charge transfer from the metal to the σ* orbital of .

**Figure 5:**
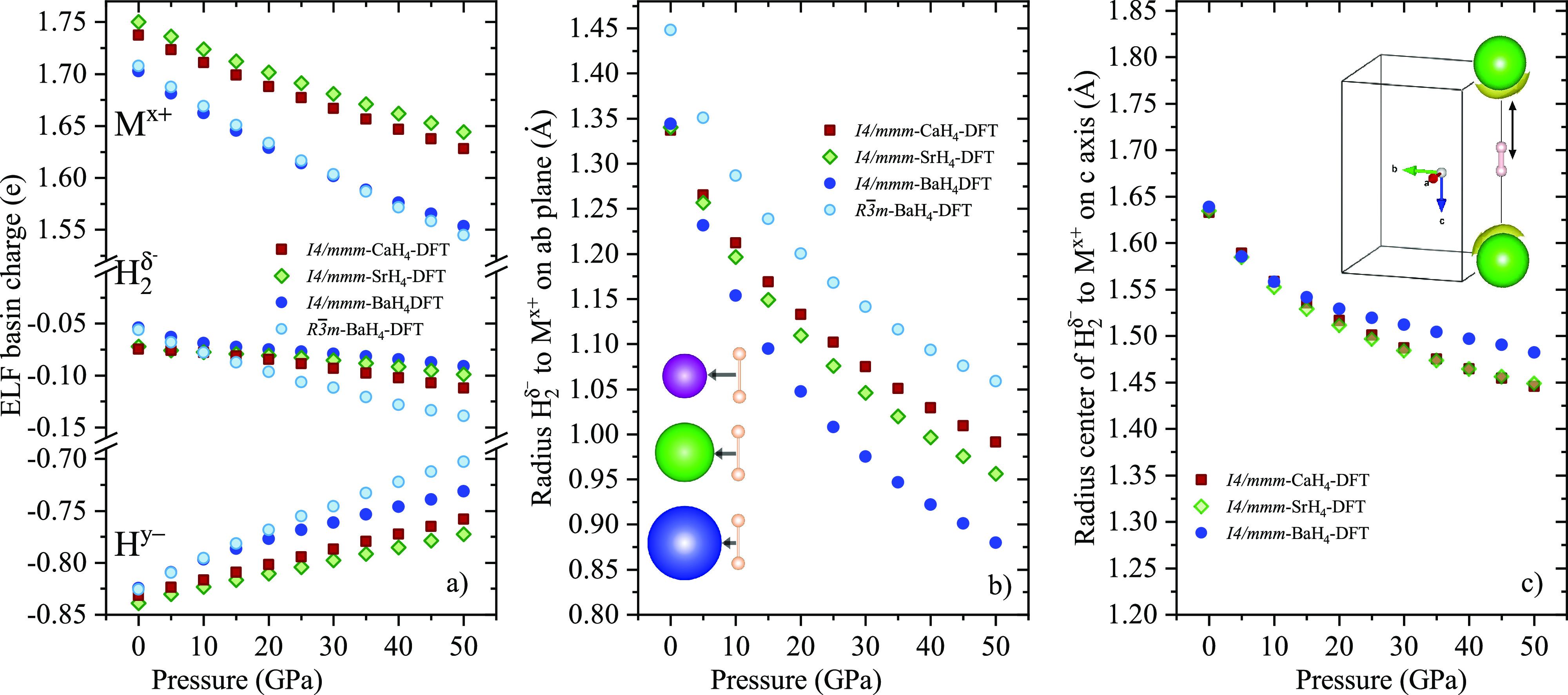
(a) Charges as a function of pressure based on the ELF topology of the metals (M^*x*+^, *x*+ is the charge), hydrogen atoms located in the tetrahedral sites (H^*y*–^, *y*– is the charge) and molecular hydrogen located in the octahedral sites (, δ – is the charge). b) Radius of the  ELF basin along the line joining its middle point and the alkaline-earth metals across the *I*4/*mmm ab* plane, and the octahedral diagonal in . Insert: A metallic atom of the *I*4/*mmm ab* plane corner hosting one  in the center perpendicular to the plane, blue is for Ba, green for Sr and purple for Ca, H_2_ molecule is pink. c) Radius of the  ELF basin across the *I*4/*mmm c* axis. Insert: *I*4/*mmm c* axis with 2 metallic atoms on the corners hosting one  in the center along the axis. The black arrows represent the  ELF basin radius.

The topological analysis of the ELF can offer further insight into the chemical origin of the  frequency downshift and associated bond elongation.^52^ In particular, we explore the ELF basin radius of the  molecule along the line joining its middle point and the alkaline-earth atoms (see Figure 5, parts b and c). The *I*4/*mmm* structures have two ELF  radii to consider because of the pseudo-octahedral metallic coordination, one along the *ab* plane and one along the *c* axis (see Figure S10). The  (used to model the experimental  BaH_4_, Figure S10) has only one Ba-to- and therefore only one radius of the ELF basin of the  molecule along the line joining its middle point in the pseudo-octahedral metallic coordination. This  BaH_4_ Ba-to- distance is longer than the Ba-to- distance in the *I*4/*mmm ab* plane, but shorter than that in the *I*4/*mmm c* axis. Consequently, the resultant compression of the  radius for the six equidistant  Ba atoms is longer, justifying the similar Raman downshift of the vibron observed in  compared to *I*4/*mmm* BaH_4_. Upon compression of the *I*4/*mmm* structure, the  ELF basin radius in the *ab* plane decreases as the alkaline-earth atomic number increases (Figure 5b). Similarly, the ELF basin radius of the  along the *c* axis also decreases upon compression, Figure 5c. We notice that, along the *c* axis, this distance is longer in BaH_4_ than in CaH_4_ and SrH_4_ (Figure 5b). As shown by the *c*/*a* ratio, *c* axis is not as compressible as *a* (Figure 1). The shorter  radius on the *ab* plane can be associated with a higher compression of the metals on that plane. As a result, there is an elongation of the  bond along the *c* axis which increases with the atomic number. This correlates with the greater vibron downshift in BaH_4_ than for CaH_4_. Within the *I*4/*mmm* structures the  unit lies along the *c* axis. Therefore, the upshift of the libron can be related to the increase in the *c*/*a* ratio in compression and with the atomic number of the cation. The larger the steric effect of the metal toward the , the more hindered the libration and the more upshifted the libron is.

In summary, our synchrotron X-ray diffraction measurements identify *I*4/*mmm* as a common structure adopted by the alkaline earth tetrahydrides, and all contain quasi-molecular hydrogen units within octahedral interstices. Comparison between the Raman spectra of the *I*4/*mmm* tetrahydrides demonstrates that the heavier alkaline earth cation causes a more pronounced downshift of the vibron Raman frequency, associated with a lengthening of the molecular hydrogen bond. Through the topological analysis of the ELF we find that the steric effect of the metal on the H–H bond is the dominating factor triggering the H–H bond lengthening. Although BaH_4_ has the largest lattice parameters, its  ELF basin radius toward the alkaline-earth metal is the smallest along the ab plane, so the H_2_ is more strongly confined than in Ca or Sr. We propose that the H_2_ bond elongation is caused by H_2_ confinement in interstitial sites of the metals tearing apart the enclosed H_2_ molecule.

## Supporting Information Available

Experimental and theoretical methods, characterization data for all new compounds, including unit-cell parameters, band structures, X-ray diffraction patterns, Raman of  BaH_4_, and theoretical Raman frequencies, and Bader and ELF topological analyses (PDF)
Transparent Peer Review report available (PDF)

## References

[ref1] Wigner E.; Huntington H. B. On the possibility of a metallic modification of hydrogen. J. Chem. Phys. 1935, 3, 764–770. 10.1063/1.1749590.

[ref2] Ashcroft N. W. Metallic hydrogen: A high-temperature superconductor?. Phys. Rev. Lett. 1968, 21, 1748 10.1103/PhysRevLett.21.1748. 15169525

[ref3] Howie R. T.; Guillaume C. L.; Scheler T.; Goncharov A. F.; Gregoryanz E. Mixed molecular and atomic phase of dense hydrogen. Phys. Rev. Lett. 2012, 108, 125501 10.1103/PhysRevLett.108.125501. 22540596

[ref4] Eremets M. I.; Troyan I. A. Conductive dense hydrogen. Nat. Mater. 2011, 10, 927–931. 10.1038/nmat3175. 22081083

[ref5] Loubeyre P.; Occelli F.; LeToullec R. Optical studies of solid hydrogen to 320 GPa and evidence for black hydrogen. Nature 2002, 416, 613–617. 10.1038/416613a. 11948345

[ref6] Gregoryanz E.; Ji C.; Dalladay-Simpson P.; Li B.; Howie R. T.; Mao H.-K. Everything you always wanted to know about metallic hydrogen but were afraid to ask. Matter Radiat. Extrem. 2020, 5, 038101 10.1063/5.0002104.

[ref7] Loubeyre P.; Occelli F.; Dumas P. Synchrotron infrared spectroscopic evidence of the probable transition to metal hydrogen. Nature 2020, 577, 631–635. 10.1038/s41586-019-1927-3. 31996819

[ref8] Dalladay-Simpson P.; Howie R. T.; Gregoryanz E. Evidence for a new phase of dense hydrogen above 325 gigapascals. Nature 2016, 529, 63–67. 10.1038/nature16164. 26738591

[ref9] Eremets M. I.; Drozdov A. P.; Kong P.; Wang H. Semimetallic molecular hydrogen at pressure above 350 GPa. Nat. Phys. 2019, 15, 1246–1249. 10.1038/s41567-019-0646-x.

[ref10] Ashcroft N. W. Hydrogen Dominant Metallic Alloys: High Temperature Superconductors ?. Phys. Rev. Lett. 2004, 92, 187002 10.1103/PhysRevLett.92.187002. 15169525

[ref11] Wang H.; Tse J.; Tanaka K.; Iitaka T.; Ma Y. Superconductive sodalite-like clathrate calcium hydride at high pressures. Proc. Natl. Acad. Sci. U. S. A. 2012, 109, 6463–6466. 10.1073/pnas.1118168109. 22492976 PMC3340045

[ref12] Bi T.; Zurek E. Electronic structure and superconductivity of compressed metal tetrahydrides. Chem. Eur. J. 2021, 27, 14858–14870. 10.1002/chem.202102679. 34469606

[ref13] Somayazulu M.; Ahart M.; Mishra A. K.; Geballe Z. M.; Baldini M.; Meng Y.; Struzhkin V. V.; Hemley R. J. Evidence for superconductivity above 260 K in lanthanum superhydride at megabar pressures. Phys. Rev. Lett. 2019, 122, 027001 10.1103/PhysRevLett.122.027001. 30720326

[ref14] Drozdov A.; Kong P.; Minkov V.; Besedin S.; Kuzovnikov M.; Mozaffari S.; Balicas L.; Balakirev F.; Graf D.; Prakapenka V.; et al. Superconductivity at 250 K in lanthanum hydride under high pressures. Nature 2019, 569, 528–531. 10.1038/s41586-019-1201-8. 31118520

[ref15] Peng F.; Sun Y.; Pickard C. J.; Needs R. J.; Wu Q.; Ma Y. Hydrogen clathrate structures in rare earth hydrides at high pressures: possible route to room-temperature superconductivity. Phys. Rev. Lett. 2017, 119, 107001 10.1103/PhysRevLett.119.107001. 28949166

[ref16] Ji C.; Li B.; Liu W.; Smith J. S.; Majumdar A.; Luo W.; Ahuja R.; Shu J.; Wang J.; Sinogeikin S.; et al. Ultrahigh-pressure isostructural electronic transitions in hydrogen. Nature 2019, 573, 558–562. 10.1038/s41586-019-1565-9. 31554980

[ref17] Borgschulte A.; Terreni J.; Billeter E.; Daemen L.; Cheng Y.; Pandey A.; Łodziana Z.; Hemley R. J.; Ramirez-Cuesta A. J. Inelastic neutron scattering evidence for anomalous H–H distances in metal hydrides. Proc. Natl. Acad. Sci. U. S. A. 2020, 117, 4021–4026. 10.1073/pnas.1912900117. 32029594 PMC7049121

[ref18] Struzhkin V. V.; Kim D. Y.; Stavrou E.; Muramatsu T.; Mao H.-k.; Pickard C. J.; Needs R. J.; Prakapenka V. B.; Goncharov A. F. Synthesis of sodium polyhydrides at high pressures. Nat. Commun. 2016, 7, 12267 10.1038/ncomms12267. 27464650 PMC4974473

[ref19] Wu G.; Huang X.; Xie H.; Li X.; Liu M.; Liang Y.; Duan D.; Li F.; Liu B.; Cui T. Unexpected calcium polyhydride CaH_4_: A possible route to dissociation of hydrogen molecules. J. Chem. Phys. 2019, 150, 044507 10.1063/1.5053650. 30709245

[ref20] Mishra A. K.; Muramatsu T.; Liu H.; Geballe Z. M.; Somayazulu M.; Ahart M.; Baldini M.; Meng Y.; Zurek E.; Hemley R. J. New Calcium Hydrides with Mixed Atomic and Molecular Hydrogen. J. Phys. Chem. C 2018, 122, 19370–19378. 10.1021/acs.jpcc.8b05030.

[ref21] Zhang C.; Chen X.-J.; Zhang R.-Q.; Lin H.-Q. Chemical trend of pressure-induced metallization in alkaline earth hydrides. J. Phys. Chem. C 2010, 114, 14614–14617. 10.1021/jp103968c.

[ref22] Tse J.; Klug D.; Desgreniers S.; Smith J.; Flacau R.; Liu Z.; Hu J.; Chen N.; Jiang D. Structural phase transition in CaH_2_ at high pressures. Phys. Rev. B 2007, 75, 134108 10.1103/PhysRevB.75.134108.

[ref23] Smith J. S.; Desgreniers S.; Klug D. D.; Tse J. High-density strontium hydride: An experimental and theoretical study. Solid State Commun. 2009, 149, 830–834. 10.1016/j.ssc.2009.03.021.

[ref24] Kinoshita K.; Nishimura M.; Akahama Y.; Kawamura H. Pressure-induced phase transition of BaH_2_: Post Ni_2_In phase. Solid State Commun. 2007, 141, 69–72. 10.1016/j.ssc.2006.09.045.

[ref25] Smith J. S.; Desgreniers S.; Tse J.; Klug D. D. High-pressure phase transition observed in barium hydride. J. Appl. Phys. 2007, 102, 043520 10.1063/1.2772427.

[ref26] Tse J.; Song Z.; Yao Y.; Smith J. S.; Desgreniers S.; Klug D. D. Structure and electronic properties of BaH_2_ at high pressure. Solid State Commun. 2009, 149, 1944–1946. 10.1016/j.ssc.2009.07.044.

[ref27] Ma L.; Wang K.; Xie Y.; Yang X.; Wang Y.; Zhou M.; Liu H.; Yu X.; Zhao Y.; Wang H.; et al. High-temperature superconducting phase in clathrate calcium hydride CaH_6_ up to 215 K at a pressure of 172 GPa. Phys. Rev. Lett. 2022, 128, 167001 10.1103/PhysRevLett.128.167001. 35522494

[ref28] Li Z.; He X.; Zhang C.; Wang X.; Zhang S.; Jia Y.; Feng S.; Lu K.; Zhao J.; Zhang J.; et al. Superconductivity above 200 K discovered in superhydrides of calcium. Nat. Commun. 2022, 13, 2863 10.1038/s41467-022-30454-w. 35606357 PMC9126910

[ref29] Semenok D. V.; Chen W.; Huang X.; Zhou D.; Kruglov I. A.; Mazitov A. B.; Galasso M.; Tantardini C.; Gonze X.; Kvashnin A. G.; et al. Sr-Doped Superionic Hydrogen Glass: Synthesis and Properties of SrH_22_. Adv. Mater. 2022, 34, 2200924 10.1002/adma.202200924. 35451134

[ref30] Peña Alvarez M.; Binns J.; Martinez-Canales M.; Monserrat B.; Ackland G. J.; Howie R. T.; Pickard C.; Gregoryanz E.; et al. Synthesis of Weaire-Phelan barium polyhydride. J. Phys. Chem. Lett. 2021, 12, 4910–4916. 10.1021/acs.jpclett.1c00826. 34008402

[ref31] Chen W.; Semenok D. V.; Kvashnin A. G.; Huang X.; Kruglov I. A.; Galasso M.; Song H.; Duan D.; Goncharov A. F.; Prakapenka V. B.; et al. Synthesis of molecular metallic barium superhydride: pseudocubic BaH_12_. Nature Commun. 2021, 12, 273 10.1038/s41467-020-20103-5. 33431840 PMC7801595

[ref33] Hooper J.; Terpstra T.; Shamp A.; Zurek E. Composition and constitution of compressed strontium polyhydrides. J. Phys. Chem. C 2014, 118, 6433–6447. 10.1021/jp4125342.

[ref34] Nylén J.; Sato T.; Soignard E.; Yarger J. L.; Stoyanov E.; Häussermann U. Thermal decomposition of ammonia borane at high pressures. J. Chem. Phys. 2009, 131, 104506 10.1063/1.3230973.

[ref35] Pickard C. J.; Needs R. Structures at high pressure from random searching. Phys. Status Solidi B 2009, 246, 536–540. 10.1002/pssb.200880546.

[ref36] Savin A.; Nesper R.; Wengert S.; Fässler T. F. ELF: The electron localization function. Angew. Chem., Int. Ed. Engl. 1997, 36, 1808–1832. 10.1002/anie.199718081.

[ref37] Herzberg G. In Molecular spectra and molecular structure; van Nostrand D., Ed.; 1945; p 457.

[ref38] Worlton T. G.; Warren J. L. Group-theoretical analysis of lattice vibrations. Comput. Phys. Commun. 1972, 3, 88–117. 10.1016/0010-4655(72)90058-6.

[ref39] Cooke P. I.; Magdău I. B.; Ackland G. J. Calculating the Raman Signal Beyond Perturbation Theory for a Diatomic Molecular Crystal. arXiv 2022; arXiv:2202.09604.

[ref40] Peña-Alvarez M.; Afonina V.; Dalladay-Simpson P.; Liu X.-D.; Howie R. T.; Cooke P. I.; Magdau I. B.; Ackland G. J.; Gregoryanz E. Quantitative rotational to librational transition in dense H_2_ and D_2_. J. Phys. Chem. Lett. 2020, 11, 6626–6631. 10.1021/acs.jpclett.0c01736. 32674573

[ref41] Hanfland M.; Hemley R. J.; Mao H.-k. Novel infrared vibron absorption in solid hydrogen at megabar pressures. Phys. Rev. Lett. 1993, 70, 3760 10.1103/PhysRevLett.70.3760. 10053955

[ref42] Hanfland M.; Hemley R.; Mao H.; Williams G. Synchrotron infrared spectroscopy at megabar pressures: Vibrational dynamics of hydrogen to 180 GPa. Phys. Rev. Lett. 1992, 69, 1129 10.1103/PhysRevLett.69.1129. 10047130

[ref43] Loubeyre P.; Jean-Louis M.; Silvera I. F. Density dependence of the intramolecular distance in solid H_2_: A spectroscopic determination. Phys. Rev. B 1991, 43, 10191 10.1103/PhysRevB.43.10191. 9996736

[ref44] Moshary F.; Chen N. H.; Silvera I. F. Pressure dependence of the vibron in H_2_, HD, and D_2_: Implications for inter-and intramolecular forces. Phys. Rev. B 1993, 48, 12613 10.1103/PhysRevB.48.12613. 10007630

[ref45] Meier T.; Laniel D.; Pena-Alvarez M.; Trybel F.; Khandarkhaeva S.; Krupp A.; Jacobs J.; Dubrovinskaia N.; Dubrovinsky L. Nuclear spin coupling crossover in dense molecular hydrogen. Nat. Commun. 2020, 11, 6334 10.1038/s41467-020-19927-y. 33303751 PMC7728769

[ref46] Magdău I. B.; Ackland G. J. Identification of high-pressure phases III and IV in hydrogen: Simulating Raman spectra using molecular dynamics. Phys. Rev. B 2013, 87, 174110 10.1103/PhysRevB.87.174110.

[ref47] Ackland G. J.; Loveday J. S. Structures of solid hydrogen at 300 K. Phys. Rev. B 2020, 101, 094104 10.1103/PhysRevB.101.094104.

[ref48] aBader R. F. W. Atoms in Molecules: A Quantum Theory; International series of monographs on chemistry, 22, Oxford University Press: Oxford, U.K., 1994.

[ref49] Hubner J.-M.; Akselrud L.; Schnelle W.; Bobnar M.; Prots Y.; Grin Y.; Schwarz U.; et al. High-Pressure Synthesis and Chemical Bonding of Barium Trisilicide BaSi_3_. Materials 2019, 12, 145 10.3390/ma12010145. PMC6337167 30621176

[ref50] Rahm M.; Cammi R.; Ashcroft N.; Hoffmann R. Squeezing all elements in the periodic table: electron configuration and electronegativity of the atoms under compression. J. Am. Chem. Soc. 2019, 141, 10253–10271. 10.1021/jacs.9b02634. 31144505

[ref51] Tantardini C.; Oganov A. R. Thermochemical electronegativities of the elements. Nat. Commun. 2021, 12, 2087 10.1038/s41467-021-22429-0. 33828104 PMC8027013

[ref52] Otero-de-la Roza A.; Johnson E.; Luana V. Critic2: A program for real-space analysis of quantum chemical interactions in solids. Comput. Phys. Commun. 2014, 185, 1007 10.1016/j.cpc.2013.10.026.

