# Supplementary material for: Chemically Assisted Precompression of Hydrogen Molecules in Alkaline-Earth Tetrahydrides
Source: J Phys Chem Lett. 2022 Sep 2;13(36):8447–54. doi: 10.1021/acs.jpclett.2c02157 (PMC9488899; doi:10.1021/acs.jpclett.2c02157)
Supplement: Supplementary file 1 — jz2c02157_si_001.pdf [file jz2c02157_si_001.pdf]

Supplementary Material for: Chemically-Assisted Pre-Compression of Hydrogen Molecules in Alkaline-Earth Tetrahydrides

Miriam Peña-Alvarez,[*,†] Jack Binns,[a,‡] Miriam Marqués,[†]
Mikhail A. Kuzovnikov,[†] Philip Dalladay-Simpson,[¶] Chris J. Pickard,[§,‖]
Graeme J. Ackland,[⊥] Eugene Gregoryanz,[†,¶,#] and Ross T. Howie[¶,†]

†*Centre for Science at Extreme Conditions and School of Physics and Astronomy, University of Edinburgh, Edinburgh EH9 3FD, United Kingdom*

‡ *Center for High Pressure Science and Technology Advanced Research, Shanghai 100094, P.R. China*

¶*Center for High Pressure Science and Technology Advanced Research, Shanghai 100094, P.R. China*

§*Department of Materials Science and Metallurgy, University of Cambridge, Cambridge CB3 0FS, United Kingdom*

‖ *Advanced Institute for Materials Research, Tohoku University, Sendai 980-8577, Japan*

⊥*Centre for Science at Extreme Conditions and School of Physics and Astronomy, University of Edinburgh, Edinburgh, U.K.*

\# *Key Laboratory of Materials Physics, Institute of Solid State Physics, Hefei 230031, P.R.China*

E-mail: mpenaal@ed.ac.uk

[a]Present address: School of Science, RMIT University, Melbourne, Victoria 3000, Australia.

METHODS

Experimental details

Elemental Ca (99.9%, Alfa Aesar), Sr (99.99%, Sigma Aldrich), Ba (99 %,Sigma Aldrich) (or BaH_2 (99.5 %, ABCR)) were loaded into diamond anvil cells in an argon atmosphere glove box and and subsequently gas loaded with research grade hydrogen (99.9995 %) at 0.2 GPa. In one of our experiments Sr was loaded with ammonia borane (NH_3BH_3) as a hydrogen source.[1] A small chip of ruby or gold was included as a pressure calibrant[2,3] and cross-checked against the vibron frequency of pure H_2, the Raman edge of stressed diamond[4] and the known equation of state of the dihydride precursor.[5,6] Ultra low-fluorescent diamonds with culet diameters ranging from 50-200 μm were used with rhenium gaskets. Samples were heated uniaxially *in situ* by directly coupling them to an yttrium-aluminum-garnet (YAG) continuous wave laser with wavelength λ =1064 nm. Collected emission spectra were fitted using a two-parameter grey-body Planck distribution.[7]

Angle-dispersive X-ray diffraction patterns were recorded at 16-ID-B, Advanced Photon Soure, United States, ID15B at the European Synchrotron RadiationFacility, France and P02.2 Petra-III, Germany. Incident beam energies in the range of 25–37 keV were used. The calibration of detector position, primary processing, azimuthal integration and background subtraction were performed using the DIOPTAS v0.5.5 software.[8] Le Bail refinements[9] were carried out in JANA2006[10] and for Rietveld refinements the POWDERCELL 2.4 program was used.[11]

Raman spectroscopy measurements were conducted using 514.5 nm excitation wavelengths via a custom-built micro-focused Raman system provided with 1800 gr/mm and 600 gr/mm gratings and a Pylon 1340x100BRX liquid nitrogen cooled camera.[12]

Computational details

Density functional theory calculations were performed with the CASTEP code[13] using the Perdew-Burke-Ernzerhof (PBE) exchange-correlation functional.[14] Geometry optimization calculations of

the tetrahydride structures were carried out using ultrasoft pseudopotentials generated by CASTEP on-the-fly. A cutoff energy of 700 eV and a k-point grid spacing better than 0.04 $Å^{-1}$ gave well converged energies and forces for all the structures.

Other exchange-correlation functionals were tested: BLYP[15] and,the semi-empirical dispersion correction (SEDC) method of Tkatchenko and Scheffler[16] to account for the van der Waals interactions. Table S1 contains the structural parameters for the three alkaline-earth metal hydrides at 25 GPa obtained with the different functionals. BLYP volumes are bigger than those obtained with the PBE functional by 3-5-4.6 %, whereas the c/a radii and intramolecular distances are lower by 1-9-2.6 % and 3.2%. The inclusion of the van der Waals contributions leads to a considerable decrease of the volume by up to 8.2 % from PBE. Experimental volumes lie between PBE and BLYP values. The inclusion of van der Waals contributions gives too low volumes and the comparison with the experiment actually become worse.

	CaH_4			SrH_4			BaH_4		
	Volume	c/a	d_{H-H}	Volume	c/a	d_{H-H}	Volume	c/a	d_{H-H}
PBE	30.67	1.653	0.782	36.15	1.653	0.782	42.95	1.6903	0.784
BLYP	31.73	1.622	0.757	37.53	1.617	0.757	44.92	1.6459	0.759
PBE+TS	28.29	1.6476	0.782	33.17	1.7199	0.791	39.48	1.7919	0.796

Table S1: Volumes per alkaline-earth metal, c/a radii and intramolecular distances for the $I4/mmm$ structures of the hydrides at 25 GPa.

Random structure searches were performed with AIRSS to explore the BaH_4 landscape. Ab initio molecular dynamics (AIMD) calculation in CASTEP were run with the NPT ensemble and with Barium assigned a fictitious mass number of 2 to more effectively sample the configuration space. A topological analysis of the electron density and the electron localization function was done with the CRITIC2 code[17] with the energy cutoff increased to 1000 eV to assure convergence. In Table S2 we present the structural parameters for $I4/mmm$ BaH_4 at 40 GPa estimated with PBE, BLYP and PBE+TS, and we included the experimental data. It can be seen that the choice of a different functional does not solve the discrepancy for c/a between theory and experiment, with an even lower c/a using BLYP.

There have been different approaches to determine the atomic charges in crystals. Probably,

	PBE	BLYP	PBE+TS	exp
Volume	38.27	39.86	35.44	38.8393
c/a	1.740	1.698	1.780	2.167
d_{H-H}	0.792	0.763	0.800	0.77

Table S2: Volumes per formula unit, c/a radii and intramolecular H-H covalent bond length for the $I4/mmm$ BaH_4 at 40 GPa.

Compound	Lattice parameters (Å)	Atoms	x	y	z
CaH_4 ($I4/mmm$)	a=b=3.129 c=5.331	Ca(2*a*)	0.0000	0.0000	0.0000
50 GPa	α=β=γ=90°	H(4*d*)	0.0000	0.5000	0.2500
		H(4*e*)	0.0000	0.0000	0.4258
SrH_4 ($I4/mmm$)	a=b=3.298 c=5.641	Sr(2*a*)	0.0000	0.0000	0.0000
50 GPa	α=β=γ=90°	H(4*d*)	0.0000	0.5000	0.2500
		H(4*e*)	0.0000	0.0000	0.4298
BaH_4 ($I4/mmm$)	a=b=3.445 c=6.074	Ba(2*a*)	0.0000	0.0000	0.0000
50 GPa	α=β=γ=90°	H(4*d*)	0.0000	0.5000	0.2500
		H(4*e*)	0.0000	0.0000	0.4345
BaH_4 ($I4/mmm$)	a=b=2.637 c=6.492	Ba(2*a*)	0.0000	0.0000	0.0000
200 GPa	α=β=γ=90°	H(4*d*)	0.0000	0.5000	0.2500
		H(4*e*)	0.0000	0.0000	0.3156
BaH_4 ($R\bar{3}m$)	a=b=4.289 c=9.708	Ba(3*b*)	0.0000	0.0000	0.5000
10 GPa	α=β=90 γ=120°	H(6*c*)	0.0000	0.0000	0.7646
		H(6*c*)	0.0000	0.0000	0.0403

Table S3: Calculated structural parameters of the predicted $I4/mmm$ phases of CaH_4, SrH_4 and BaH_4 at 50 GPa, BaH_4 at 200 GPa and the $R\bar{3}m$ phase of BaH_4 at 10 GPa using PBE. We have calculated the ELF of the metallic sublattices (without hydrogens).

one of the most straightforward and mathematically rigorous is the Quantum Theory of Atoms in Molecules developed by Bader.[18] It is based on the topology of the electron density. It shows maxima (cusps) on the atoms and decays exponentially when moving away from them. This allows the partition of the crystalline space in topological atoms defined by the union of the electron density maxima with their attraction maxima and delimited by surfaces obeying the zero-flux condition for the electron density. Their charge can be calculated from the integration of the electron density within these regions. These regions are non-overlapping and additive, recovering the total volume of the crystal.

The electron localization function was introduced by Becke and Edgecombe in 1990 for the

P(GPa)	CaH_4	SrH_4	BaH_4
0	42.25	51.24	64.26
5	38.42	46.00	56.29
10	35.73	42.50	51.33
15	33.68	39.89	47.82
20	32.04	37.84	45.12
25	30.67	36.15	42.95
30	29.50	34.73	41.15
35	28.49	33.50	39.61
40	27.60	32.42	38.27
45	26.81	31.47	37.17
50	26.10	30.61	36.05

Table S4: PBE calculated volume per metallic atom in the $I4/mmm$ structure for the alkaline-earth tetrahydrides versus pressure

analysis in real space of the electron localization,[19] and later reinterpreted by Savin in terms of the Pauli kinetic energy density (t_p).[20] By definition, ELF is a relative measure of the electron localization with respect to the homogenous electron gas (HEG). In general, the ELF value approaches 1 in regions of the space where electron pairing occurs (e.g., atomic shells, bonds and lone pairs). In analogy with QTAIM, a partition of the space based on the ELF can be performed. It consists of non-overlapping basins with well-defined chemical interpretation (cores, bonds, lone pairs). Moreover, the basin charges come from integration of the electron density within these regions. We have also calculated the radius of the H_2 ELF basin along the line joining its middle point and the alkaline-earth metal. It corresponds to the distance from the middle point to the minimum of the ELF profile along the line connecting it to the alkaline-earth metal.

Regardless of the Ca/Sr/Ba host and structure, the positions of the hydrogens are dictated by the electronic structure of the host lattices, as we find that hydrogens in the tetrahydrides are located on the ELF maxima of the empty metallic lattices (see Fig. 2).

Because $I4/mmm$ is a subgroup of fcc and bcc, fcc Ba can be described within the $I4/mmm$ space group with cell parameters a=a$_{fcc}/\sqrt{2}$, c=a$_{fcc}$. Thus, the fcc Ba ELF topology is equivalent to that of $I4/mmm$ Ba: octahedral ELF maxima with bigger ELF values are the preferred site for the $H_2^{\delta-}$ units, whereas the H^- atoms are located on the tetrahedral sites, Fig. 2b of the main text.

	ELF_{max}(oct)		ELF_{max}(tet)	
	ELF value	ELF basin volume ($Å^3$)	ELF value	ELF basin volume ($Å^3$)
Ca ($I4/mmm$)	0.794	14.4282	0.472	1.4581
Sr ($I4/mmm$)	0.705	13.1247	0.406	2.2979
Ba ($I4/mmm$)	0.477	9.6122	0.318	2.6687
Ba ($Fm\bar{3}m$)	0.653	19.1620	0.384	5.0335

Table S5: Calculated ELF values at the octahedral and tetrahedral ELF maxima of the pure metallic sublattices (without hydrogens) of $I4/mmm$ CaH_4, SrH_4 and BaH_4 at 50 GPa and the $Fm\bar{3}m$ Ba sublattice at 10 GPa. The volumes of their corresponding basins are included in $Å^3$. We have also calculated the ELF of the metallic sublattice of the experimental BaH_4 at 50 GPa with c/a=2.17. In this case the elongated basin centered on the octahedral sites (2*b* sites) breaks into 2 separate maxima on 4*e* sites with ELF value=0.480, with the ELF value 0.336 at the maxima on tetrahedral sites (4*d* sites) . Calculated I4/mmm BaH_4 structure at 200 GPa has c/a >2.2 and becomes more stable than the low c/a I4/mmm BaH_4 structure. This structure at 200 GPa does not have any H_2 molecules as all H-H distances are above 1.5Å, and all the H_2, H-) bonds are broken. In the presence of excess hydrogen, it is calculated to be unstable with respect to Ba_8H_{46}.

Raman calculations and vibrational modes symmetry assignment are computed by CASTEP as part of the raman calculation, as implemented by Keith Refson.[21] The visualization of the vibrations within the lattice related to the Raman modes was done using Jmol.[22]

	DFPT E_{2g} $H_2^{\delta-}$ lib. $/cm^{-1}$	Exp. E_{2g} $H^{\delta-}$ lib. $/cm^{-1}$	DFPT E_{2g} $H^{\delta-}$+H^- $/cm^{-1}$	Exp. E_{2g} $H^{\delta-}$+H^- $/cm^{-1}$	DFPT A_{1g} H^- $/cm^{-1}$	Exp. A_{1g} H^- $/cm^{-1}$	DFPT A_{1g} $H^{\delta-}$ v. $/cm^{-1}$	Exp. A_{1g} $H^{\delta-}$ v. $/cm^{-1}$
CaH_4 $I4/mmm$	747	675	1355	—-	1425	—-	3606	3670
SrH_4 $I4/mmm$	852	790	1306	—-	1436	—-	3597	3600
BaH_4 $I4/mmm$	988	970	1212	1282	1445	—-	3509	3330
BaH_4 $R\bar{3}m$	1159	940	1265	1125	1311	—-	2900	3300

Table S6: Raman shift of the $I4/mmm$ tetrahydrides theoretically calculated[23] and experimentally observed. Experimental fcc BaH_4 is theoretically approached taking the $R\bar{3}m$ as template frozen H_2 molecules. DFTP was used to calculate vibrational frequencies.[23] Experimental bands labelled with —- overlap with diamond first order Raman mode and therefore are not observable. Differences between BaH_4 $I4/mmm$ theoretical and experimental are due to the different lattice parameters. Differences between $R\bar{3}m$ and fcc BaH_4 are due to structural differences and calculations not accounting for the H_2 rotation. Differences between theory and experiment are very significant in BaH_4 $I4/mmm$ because as said in the main text although the space groups are the same, the lattice parameters are different and the experimental structure is not stable. Difference between the theoretical $R\bar{3}m$ and experimental fcc are expected as the $R\bar{3}m$ is taken just as a way to mimic the cubic structure, and $R\bar{3}m$ distorts in compression.

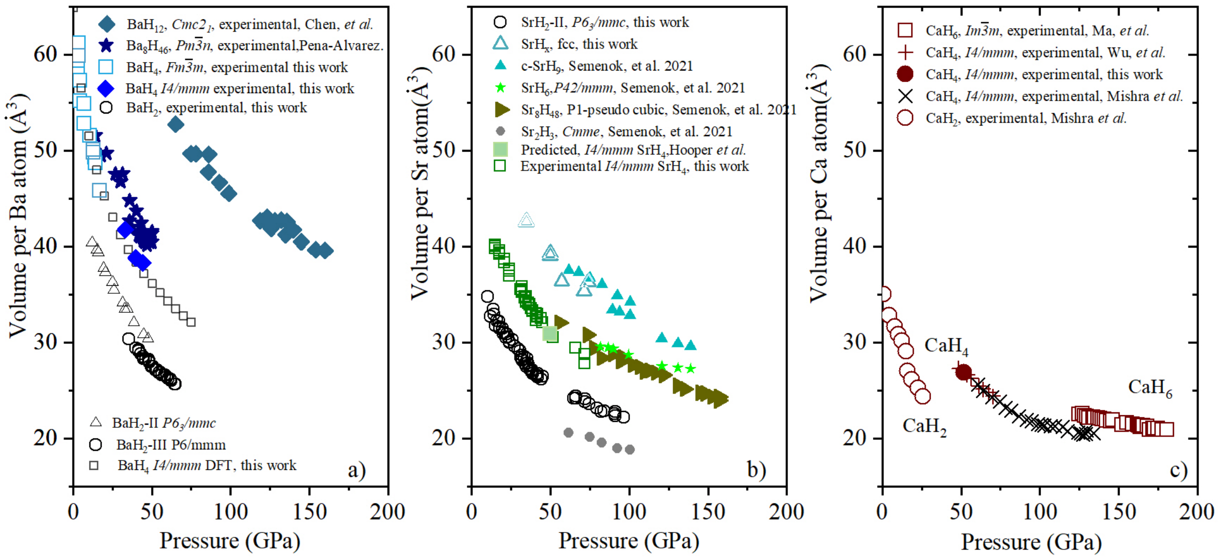

Figure S1: Volume per Ba/Sr/Ca atom as function of pressure for their corresponding hydrides. The volumes of BaH_2; BaH_4; SrH_2 and SrH_4 are from this work. BaH_{12} is taken from Chen *et al.*[24] and Ba_8H_{46} from Pena-Alvarez, *et al.*[25] Sr_2H_3, Sr_8H_{48}, SrH_6 and SrH_9 are from Semenok, *et al.*[26] compared with the empty turquoise triangle representing SrH_9 from this study. CaH_2 and CaH_4 are from Mishra, *et al.*[27] and from Wu, *et al.*[28] whilst CaH_6 is from Ma, *et al.*[29]

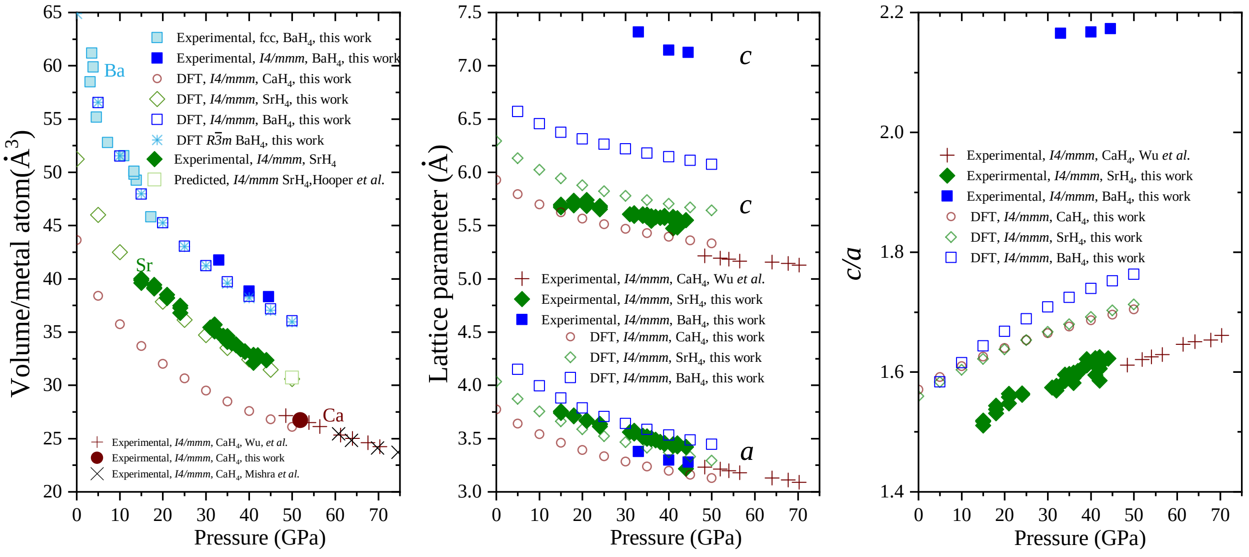

Figure S2: Comparison between experimentally derived and calculated lattice parameters for the tetrahydrides. a) Volume per Ba/Sr/Ca atom as a function of pressure. b) Lattice parameters for the $I4/mmm$ structure as a function of pressure. c) c/a ratios for the $I4/mmm$ structure as a function of pressure.

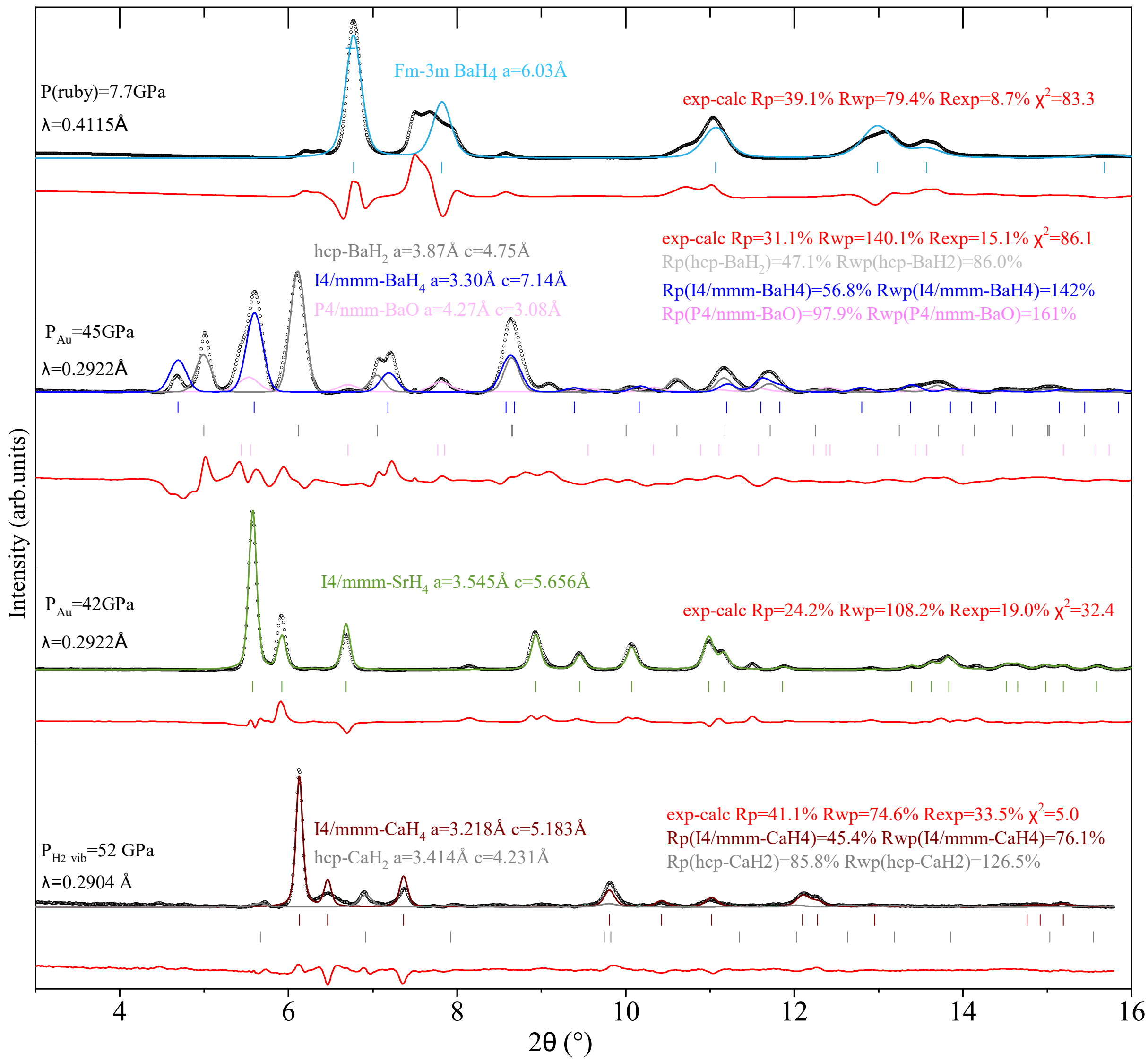

Figure S3: X-ray diffraction patterns of Ca, Sr and Ba tetrahydrides, with their Rietveld refinement shown as solid lines and the raw data as symbols. Tick marks indicate the Bragg peaks of identified phases.

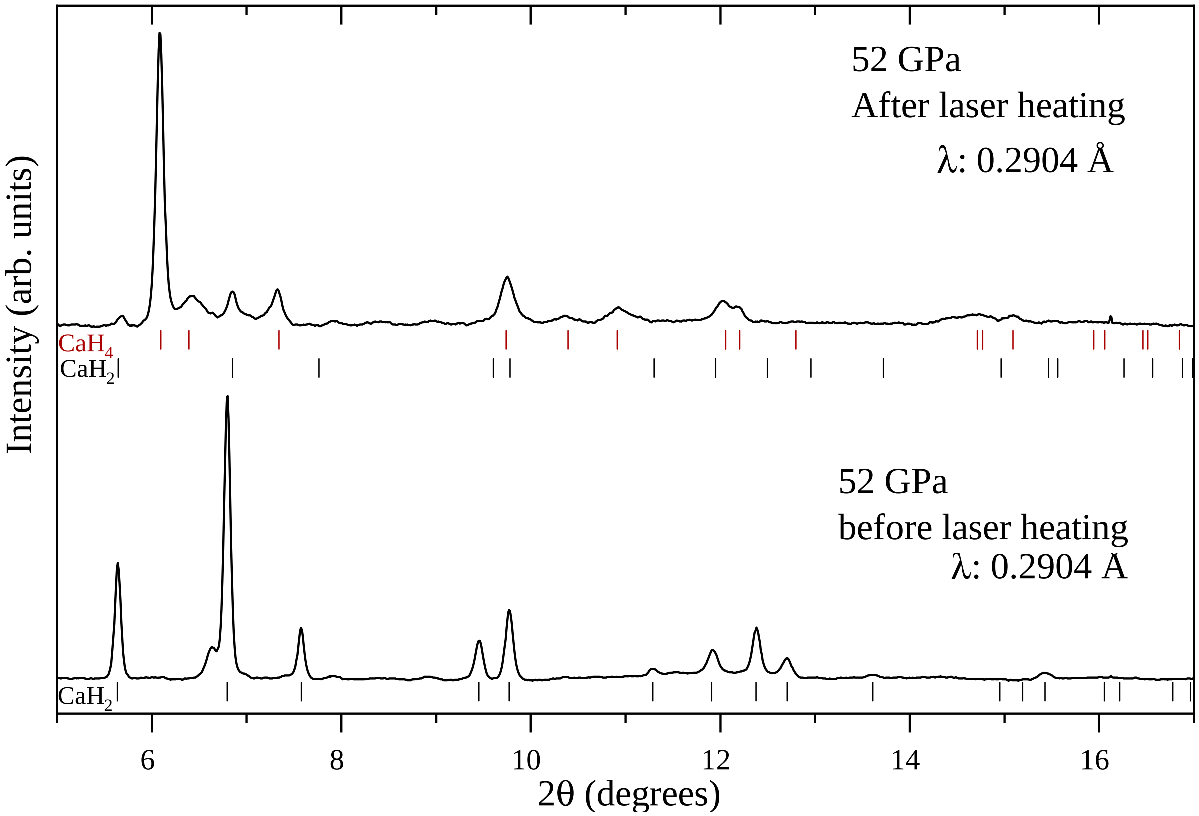

Figure S4: X-ray diffraction patterns of Calcium and hydrogen compressed to 52 GPa, before (bottom) and after laser heating (top). CaH_2 is refined to hexagonal $P6_3/mmc$ and CaH_4 is refined to tetragonal $I4/mmm$ ($\lambda = 0.2904 Å$). Tick marks indicate Bragg peaks from the labelled phases.

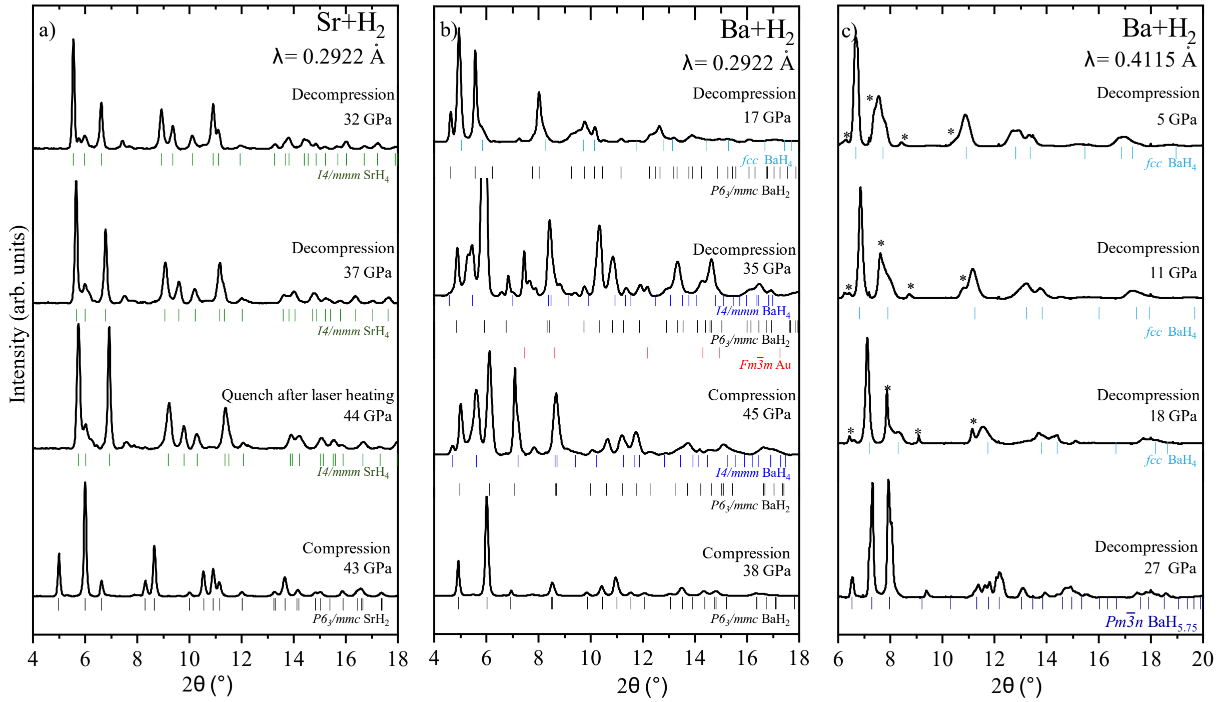

Figure S5: a) Representative X-ray diffraction patterns of strontium hydride before laser heating (43 GPa), after laser heating (44 GPa) and upon decompression. SrH_4 is refined to tetragonal $I4/mmm$ ($\lambda = 0.2922$ Å). Tick marks indicate Bragg peaks from the labelled phases. b) Representative X-ray diffraction patterns of barium hydrides. BaH_4 is refined to $I4/mmm$ upon compression to 45 GPa and on decompression to 35 GPa. At 17 GPa BaH_4 is refined to the fcc phase ($\lambda = 0.2922$ Å). c) Representative X-ray diffraction patterns of barium hydrides upon decompression from the Ba_8H_{46} phase.[25] At 18 GPa and below, the patterns are refined to fcc BaH_4($\lambda = 0.4115$ Å). Asterisks correspond to BaH_2.

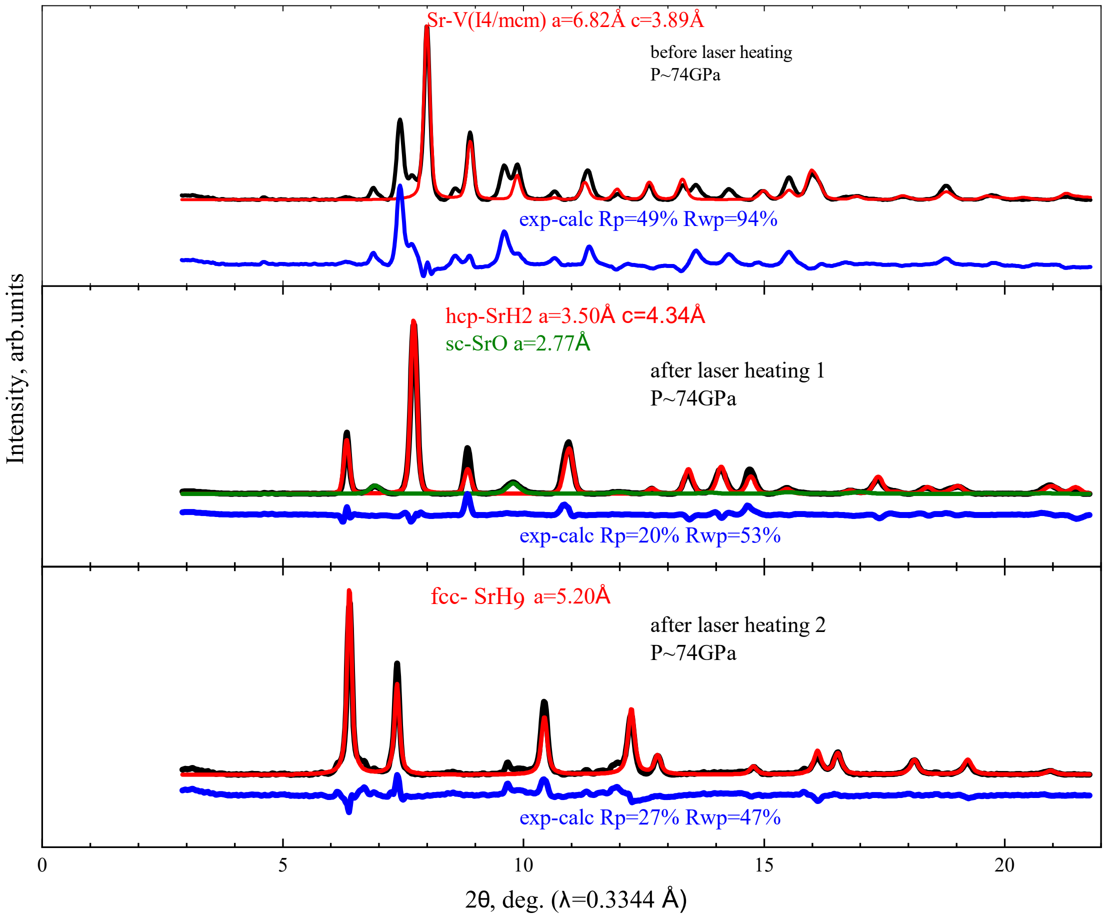

Figure S6: Rietveld refinement of the XRD patterns collected from an Sr + NH_3BH_3 experiment, collected before (top) and after (middle and bottom) laser heating at a pressure of 74 GPa. After the first laser heating SrH_2 is formed and after a subsequent laser heating the patterns can be indexed to an fcc structure of which the lattice parameters and volumes agree with the previously reported SrH_9.[26]

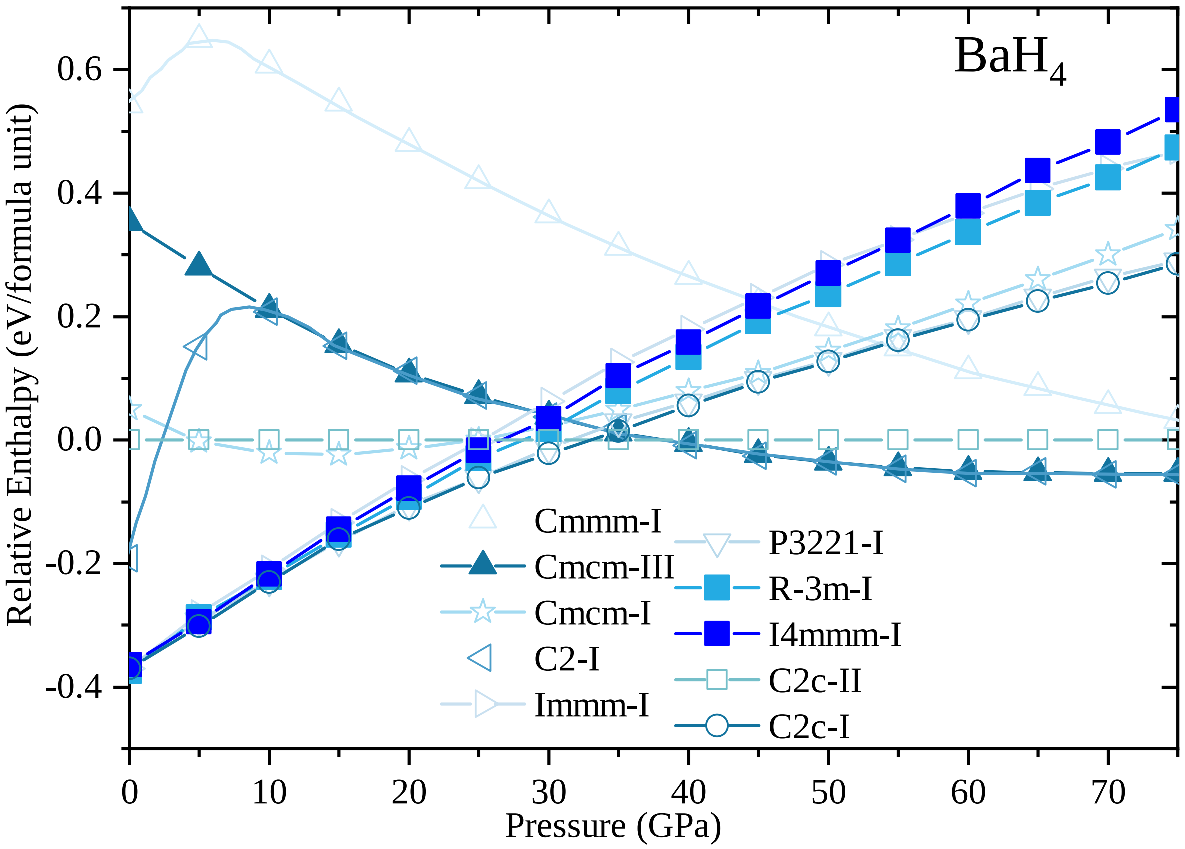

Figure S7: DFT enthalpy differences as a function of pressure for candidate structures of BaH_4 from AIRSS with different symmetries vs $C2/c-II$. The group of stable structures at lower pressures are small distortions from fcc, and are predicted to become fcc at 300K.

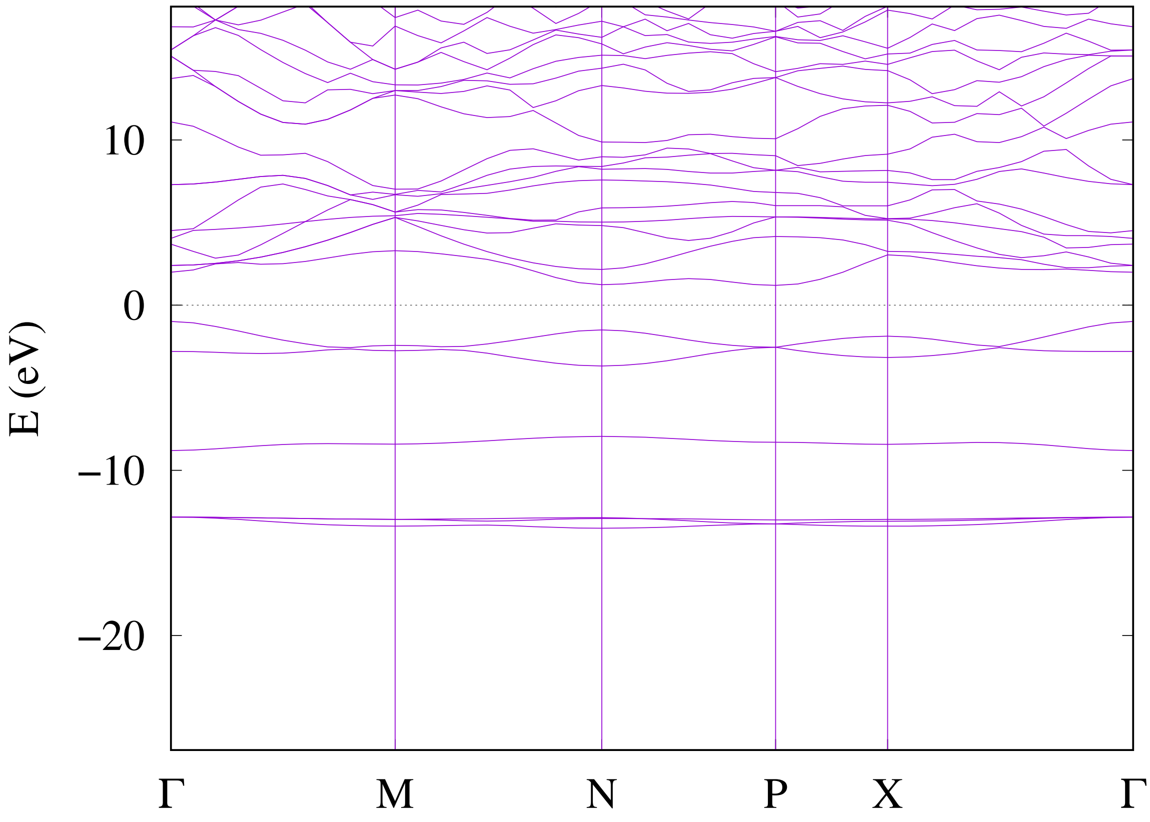

Figure S8: Band structure of $I4/mmm$ BaH_4 at 0 GPa

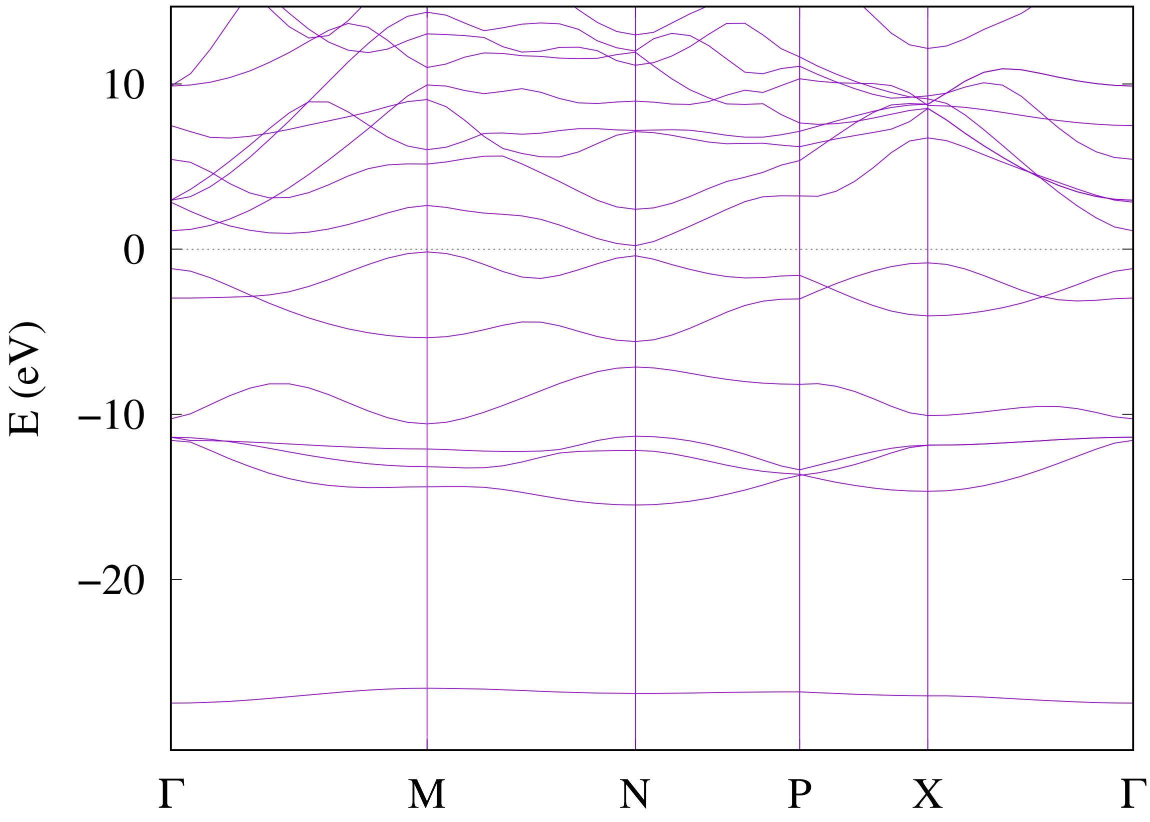

Figure S9: Band structure of $I4/mmm$ BaH_4 at 50 GPa

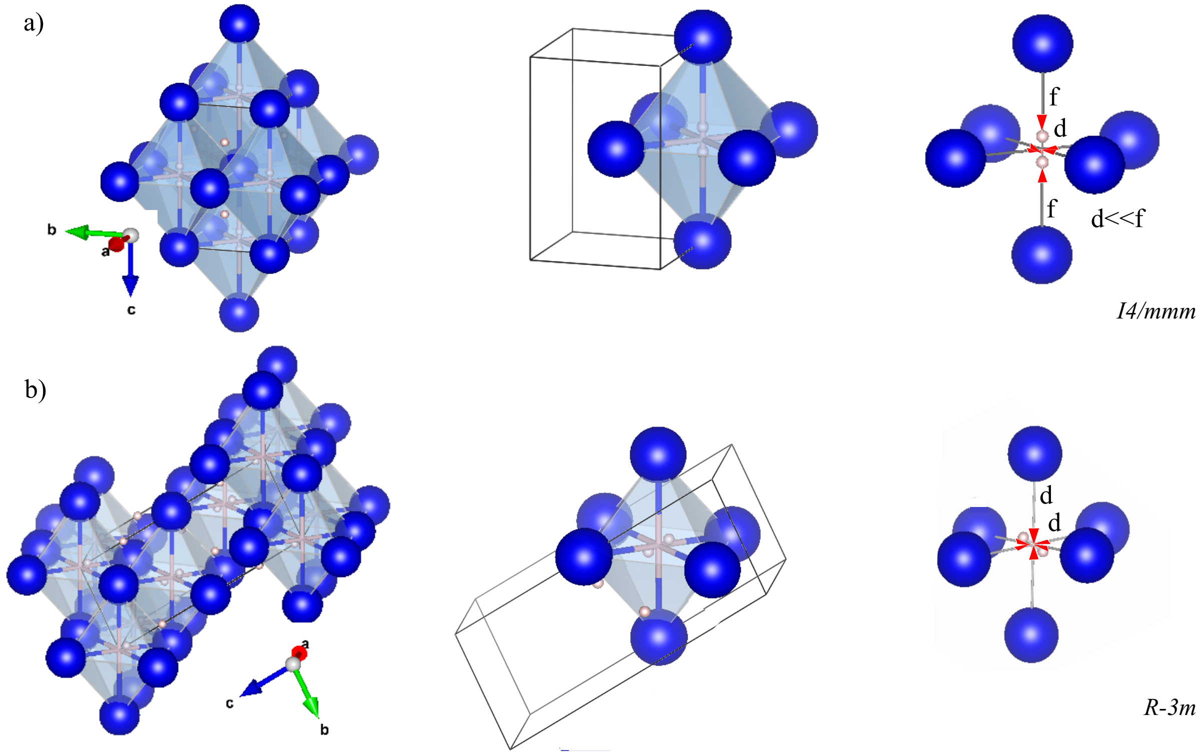

Figure S10: a) The $I4/mmm$ pseudo-octahedral metallic coordination shell of the $H_2^{\delta-}$ units (with D_{4h} symmetry). In CaH_4 and SrH_4, this can be viewed as a small distortion of the cation positions from $Fm\bar{3}m$ with the $H_2^{\delta-}$ units parallel to the c axis. The larger distortion seen for $I4/mmm$ BaH_4 ($c/a > 2$) leads to a more elongated octahedron consistent with the larger H-H distance. b) $R\bar{3}m$ BaH_4, a theoretical stable structure found in AIRSS with the molecule oriented on $(\frac{1}{2}, \frac{1}{2}, \frac{1}{2})$; this has a more regular octahedron, similar to the experimentally observed $Fm\bar{3}m$ with a higher ELF-value at the octahedral maximum than $I4/mmm$. Other stable structures found by AIRSS have similar Ba sublattices with various other molecular orientations and associated distortions of the octahedral site. With enforced $I4/mmm$ symmetry a local minimum energy structure exists with $c/a = 2.3$, no molecular hydrogens, and each Ba in a cage of 18 hydrogens. This BaH_4 $I4/mmm$ structure becomes more stable than the molecule-containing version at higher pressures around 160 GPa (Ba_8H_{46} is still the ground state).

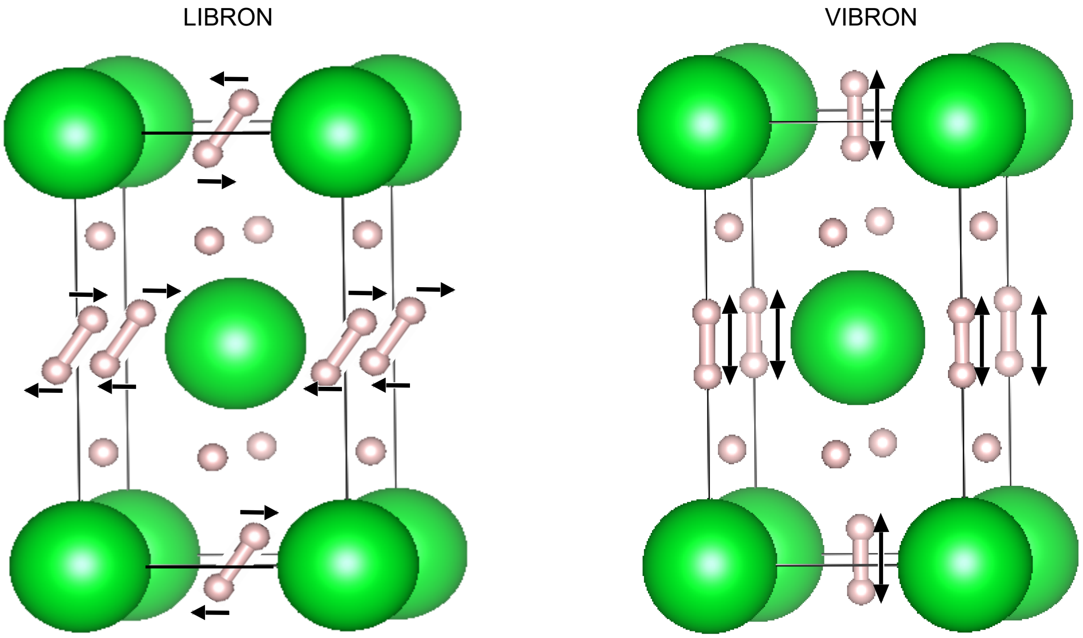

Figure S11: Schematic representation of the librational mode (left) and the H-H vibrational stretching mode (right) within $I4/mmm$ tetrahydrides.

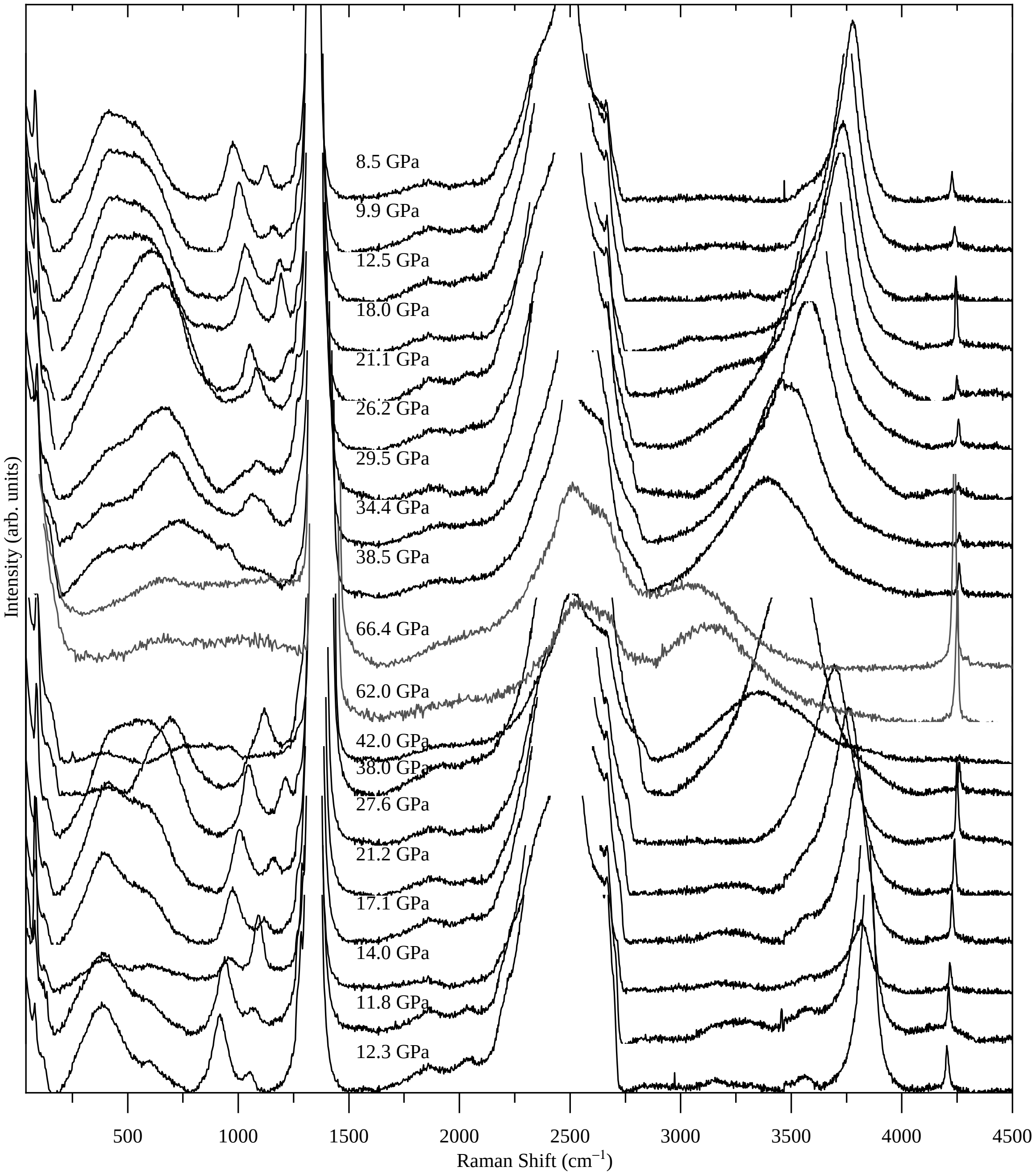

Figure S12: Representative Raman spectra for *fcc* BaH_4 + H_2 upon compression and decompression. Grey spectra correspond to selected pressures from a different experimental run.

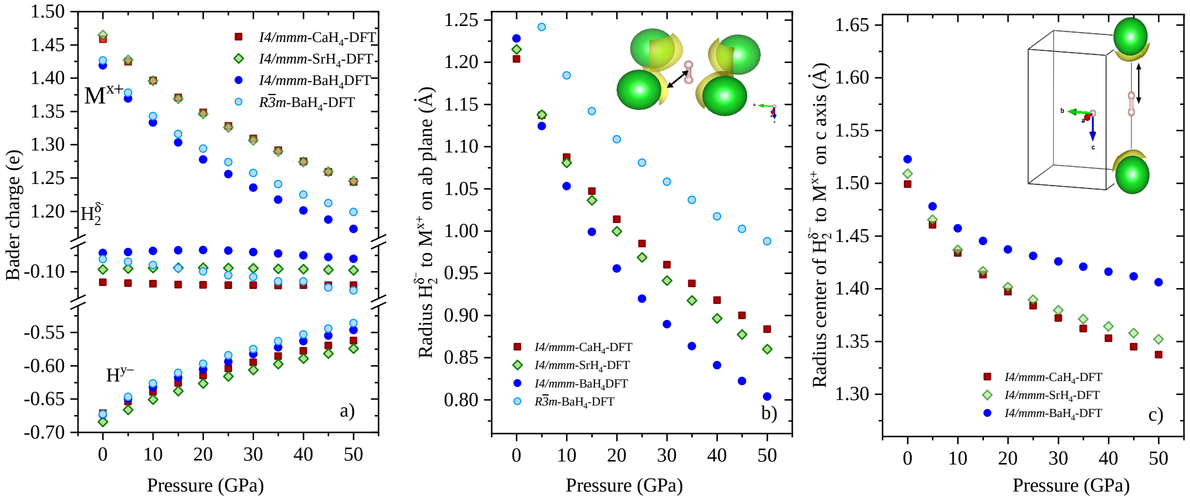

Figure S13: a) Charges of the metals (M), the hydrogens located in tetrahedral sites (H^{y-}) and of molecular hydrogen located in the octahedral sites ($H_2^{\delta-}$) based on the Bader topology. b,c) Radius of the $H_2^{\delta-}$ Bader basin within the MH_4 along the line joining its middle point and the alkaline-earth metals: b) across the $I4/mmm$ *ab* plane, and octahedral diagonal on the $R\bar{3}m$; c) across the $I4/mmm$ *c* axis. Inserted schemes show: b) the $I4/mmm$ *ab* plane with 4 metallic atoms on the corners hosting one $H_2^{\delta-}$ in the center perpendicular to the plane; c) $I4/mmm$ *c* axis with 2 metallic atoms on the corners hosting one $H_2^{\delta-}$ in the center along the axis. The black arrows represent the $H_2^{\delta-}$ Bader basin radius.

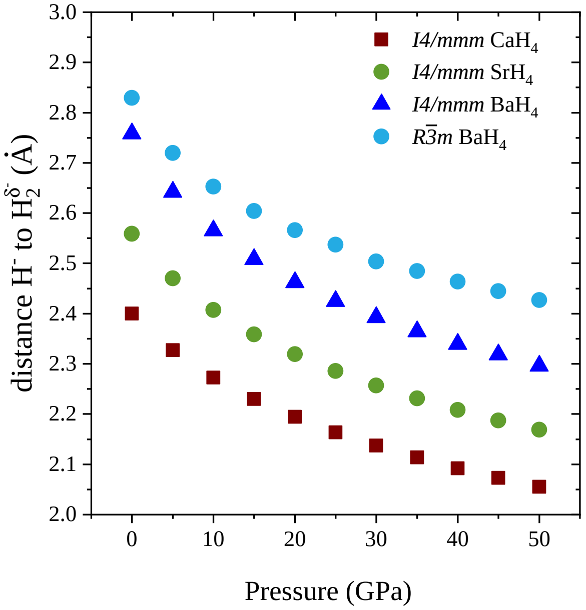

Figure S14: a) Radius of the ELF basin of the H^- along the line joining it with the $H_2^{\delta-}$ middle point.

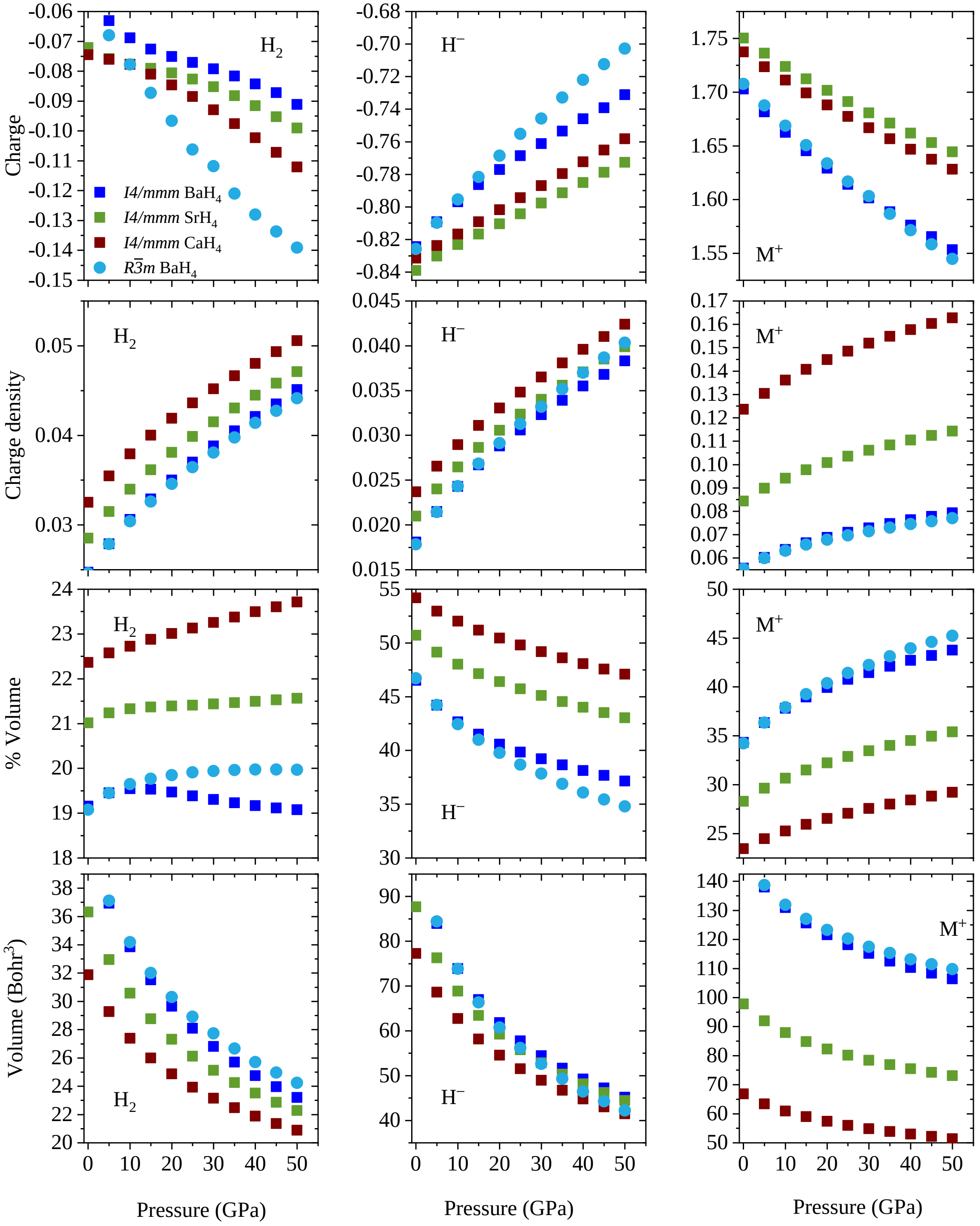

Figure S15: ELF results for the tetrahydride structures as a function of pressure, per columns from left to right is H_2, H^- and M^{2+}. Per rows, from top to bottom: Basin charge (e), Basin charge density (e/Bohr^3), % Volume, Volume.

References

(1) Nylén, J.; Sato, T.; Soignard, E.; Yarger, J. L.; Stoyanov, E.; Häussermann, U. Thermal decomposition of ammonia borane at high pressures. *J. Chem. Phys.* **2009**, *131*, 104506.

(2) Mao, H. K.; Bell, P. M.; Hemley, R. J. Ultrahigh pressures: Optical observations and Raman measurements of hydrogen and deuterium to 1.47 Mbar. *Physical Review Letters* **1985**, *55*, 99–102.

(3) Takemura, K.; Dewaele, A. Isothermal equation of state for gold with a He-pressure medium. *Physical Review B - Condensed Matter and Materials Physics* **2008**, *78*, 1–13.

(4) Howie, R. T.; Gregoryanz, E.; Goncharov, A. F. Hydrogen (deuterium) vibron frequency as a pressure comparison gauge at multi-Mbar pressures. *Journal of Applied Physics* **2013**, *114*, 073505.

(5) Smith, J. S.; Desgreniers, S.; Tse, J. S.; Klug, D. D.; Smith, J. S.; Desgreniers, S.; Tse, J. S.; Klug, D. D. High-pressure phase transition observed in barium hydride. *Journal of Applied Physics* **2007**, *043520*.

(6) Tse, J. S.; Song, Z.; Yao, Y.; Smith, J. S.; Desgreniers, S.; Klug, D. D. Structure and electronic properties of BaH_2 at high pressure. *Solid State Communications* **2009**, *149*, 1944–1946.

(7) Shen, G.; Rivers, M. L.; Wang, Y.; Sutton, S. R. Laser heated diamond cell system at the Advanced Photon Source for in situ X-ray measurements at high pressure and temperature. *Review of Scientific Instruments* **2001**, *72*, 1273–1282.

(8) Prescher, C.; Prakapenka, V. B. DIOPTAS: a program for reduction of two-dimensional X-ray diffraction data and data exploration. *High Pressure Research* **2015**, *35*, 223–230.

(9) Le Bail, A.; Duroy, H.; Fourquet, J. Ab-initio structure determination of LiSbWO 6 by X-ray powder diffraction. *Materials Research Bulletin* **1988**, *23*, 447–452.

(10) Petříček, V.; Dušek, M.; Palatinus, L. Crystallographic computing system JANA2006: general features. *Zeitschrift für Kristallographie-Crystalline Materials* **2014**, *229*, 345–352.

(11) Kraus, W.; Nolze, G. POWDER CELL–a program for the representation and manipulation of crystal structures and calculation of the resulting X-ray powder patterns. *J. Appl. Crystallogr.* **1996**, *29*, 301–303.

(12) Dalladay-Simpson, P.; Howie, R. T.; Gregoryanz, E. Evidence for a new phase of dense hydrogen above 325 gigapascals. *Nature* **2016**, *529*, 63–67.

(13) Clark, S. J.; Segall, M. D.; Pickard, C. J.; Hasnip, P. J.; Probert, M. J.; Refson, K.; Payne, M. C. First principles methods using CASTEP. *Zeitschrift fuer Kristallographie* **2005**, *220*, 567–570.

(14) Perdew, J. P.; Burke, K.; Ernzerhof, M. . *Physical Review Letters* **1996**, *77*, 3865.

(15) Lee, C.; Yang, W.; Parr, R. G. *Phys. Rev. B* **1988**, *37*, 785.

(16) Tkatchenko, A.; Scheffler, M. *Phys. Rev. Lett.* **2009**, *102*, 073005.

(17) Otero-de-la Roza, A.; Johnson, E.; Luana, V. Critic2: A program for real-space analysis of quantum chemical interactions in solids. *Comput. Phys. Commun.* **2014**, *185*, 1007.

(18) Bader, R. *Chemical Reviews* **1991**, *91*, 893.

(19) Becke, A. D.; Edgecombe, K. E. *J. Chem. Phys* **1990**, *92*, 5397.

(20) Savin, A.; Jepsen, O.; Flad, J.; Andersen, L.; Preuss, H.; von Schering, H. G. *Angew. Chem. Int. Ed. Engl.* **1992**, *31*, 187.

(21) Clark, S. J.; Segall, M. D.; Pickard, C. J.; Hasnip, P. J.; Probert, M. I.; Refson, K.; Payne, M. C. First principles methods using CASTEP. *Zeitschrift für kristallographie-crystalline materials* **2005**, *220*, 567–570.

(22) Jmol., : an open-source Java viewer for chemical structures in 3D. `http://www.jmol.org/`, Last accessed on 2022-08-05.

(23) Worlton, T. G. T.; WARREN, J. L. Group-theoretical analysis of lattice vibrations. *Computer Physics Communications* **1972**, *3*, 88–117.

(24) Chen, W.; Semenok, D. V.; Kvashnin, A. G.; Huang, X.; Kruglov, I. A.; Galasso, M.; Song, H.; Duan, D.; Goncharov, A. F.; Prakapenka, V. B. et al. Synthesis of molecular metallic barium superhydride: pseudocubic BaH_{12}. *Nature Communications* **2021**, *12*, 1–9.

(25) Peña Alvarez, M.; Binns, J.; Martinez-Canales, M.; Monserrat, B.; Ackland, G. J.; Howie, R. T.; Pickard, C.; Gregoryanz, E. Synthesis of Weaire-Phelan barium polyhydride.

(26) Semenok, D. V.; Chen, W.; Huang, X.; Zhou, D.; Kruglov, I. A.; Mazitov, A. B.; Galasso, M.; Tantardini, C.; Gonze, X.; Kvashnin, A. G. et al. Sr-Doped Superionic Hydrogen Glass: Synthesis and Properties of SrH_{22}. *Advanced Materials* **2022**, 2200924.

(27) Mishra, A. K.; Muramatsu, T.; Liu, H.; Geballe, Z. M.; Somayazulu, M.; Ahart, M.; Baldini, M.; Meng, Y.; Zurek, E.; Hemley, R. J. New Calcium Hydrides with Mixed Atomic and Molecular Hydrogen. *The Journal of Physical Chemistry C* **2018**, *122*, 19370–19378.

(28) Wu, G.; Huang, X.; Xie, H.; Li, X.; Liu, M.; Liang, Y.; Duan, D.; Li, F.; Liu, B.; Cui, T. Unexpected calcium polyhydride CaH4 : A possible route to dissociation of hydrogen molecules Unexpected calcium polyhydride CaH 4 : A possible route to dissociation of hydrogen molecules. **2019**, *150*, 044507.

(29) Ma, L.; Wang, K.; Xie, Y.; Yang, X.; Wang, Y.; Zhou, M.; Liu, H.; Yu, X.; Zhao, Y.; Wang, H. et al. High-temperature superconducting phase in clathrate calcium hydride CaH 6 up to 215 K at a pressure of 172 GPa. *Physical Review Letters* **2022**, *128*, 167001.
