# Supplementary material for: Chemically Assisted Precompression of Hydrogen Molecules in Alkaline-Earth Tetrahydrides
Source: J Phys Chem Lett. 2022 Sep 2;13(36):8447–54. doi: 10.1021/acs.jpclett.2c02157 (PMC9488899; doi:10.1021/acs.jpclett.2c02157)
Supplement: Supplementary file 2 — jz2c02157_si_002.pdf [file jz2c02157_si_002.pdf]

jz-2022-02157f.R1

Name: Peer Review Information for "Chemically-Assisted Pre-Compression of Hydrogen Molecules in Alkaline-Earth Tetrahydrides"

First Round of Reviewer Comments

Reviewer: 1

Comments to the Author

This is a beautiful excursion into the world of high-pressure chemistry/physics by Raman spectroscopy, synchrotron XRD which are carried out at pressures of up to typically 45 GPa, in some cases up to 70 GPa for tetrahydrides of Ca, Sr, and Ba. The work is nicely supplemented by extensive pressure dependent density functional theory, DFT, computations. This combination of tools allows the monitoring of the changes of the chemical bond between the hydrogen atoms and their "libration" concerning the H2 units in these crystals as a function of high pressure. Complex phase behavior is observed and interpreted by the DFT work. The weakening of the H-H bonds as manifested by the increase of the bond distance and the decrease of the H-H Raman active vibrational frequency are well characterized for the first time.

The identification of some of the Raman modes to "libration" needs to be further explained and put into context. These are presumably k=0 phonon modes. How many such modes are there and what are their symmetries? The librational motion and its relationship to rotation in high pressure H2 has been extensively studied. However, in the presented crystal structures the motions of the H2 molecules relative to the heavy ions/atoms needs further clarification. Just to say "libration" does not do full justice to what the nature of these modes are, although Fig S11 is helpful, but there are more phonon modes than shown there.

The article mentions that "The most intense band, above 3000 cm-1 (Fig. 3), corresponds to the A1g mode resulting from the H-H intramolecular stretching of the H2, vibron (see Fig. 3 and Fig. S11). How was the symmetry assigned? (See also Table S2.)

The references given in this context are not shedding light on the symmetry assignments of these modes. [19, 33, 38, 39] Can you provide the information about the symmetry assignment?

Minor points:

Can you provide figures for the relative intensities of the Raman modes as a function of pressure?

Figure 1a: Two slightly different values of the wavelength are given. Please correct.

Figure 1b: The DFT values are represented by four dashed lines. This is very nice and helpful. However, the computations provide only discreet points on these curves. Similar problems arise in Fig. S1a and others.

Please provide in the SI section a table listing the specific values (pressures and the respective volumes in order to document the DFT computations more completely.

The two curves for “DFT, I4/mmm, BaH_4”, and “DFT R3m BaH_4” seem to overlap. Use symbols that help the reader to see if this is really the case, e.g. dashed line for one, and empty triangles for the other.

Could the line for “DFT, I4/mmm , CaH_4” be continued up to 70 GPa, since the experimental data extend that far?

I recommend to avoid using the term “hydrogen bond” in the title. This is article is about bonding in dihydrogen.

Reviewer: 2

Comments to the Author

The effect of chemical and hydrostatic pressure has been the subject of intense collateral research in the analysis of hydrogen superconductors. This paper aims at providing direct insight into the issue from the analysis of the Ca-Sr-Ba family (same crystalline structure). It is thus a timely and necessary investement that was missing in the literature.

Hence, I suggest its publication under minor revisions:

-The sentence "electronic weakening of the bond by filling the antibonding orbital, sigma*, and physical weakening by conning the molecule to small interstitial locations" is slightly missformulated. Both the electronic effects (charge transfer) and confinement lead to filling of the antibonding orbital.

-More details should be given on how the authors measured the ELF radius. It would also be interesting to measure the volume of the octahedral and tetrahedral cages instead when commenting on their sizes.

-Differences in distances and c/a ratio between experiment and theory are found. Did the authors try other functionals or including dispersion? This should be commented on the method section.

Author's Response to Peer Review Comments:

We thank the referees for taking their time to provide advice and constructive criticism and for recommending the manuscript for publication. Our line-by-line responses to the referees is below.

Reviewer: 1

Comments:
This is a beautiful excursion into the world of high-pressure chemistry/physics by Raman spectroscopy, synchrotron XRD which are carried out at pressures of up to typically 45 GPa, in some cases up to 70 GPa for tetrahydrides of Ca, Sr, and Ba. The work is nicely supplemented by extensive pressure dependent density functional theory, DFT, computations. This combination of tools allows the monitoring of the changes of the chemical bond between the hydrogen atoms and their "libration" concerning the H2 units in these crystals as a function of high pressure. Complex phase behavior is observed and interpreted by the DFT work. The weakening of the H-H bonds as manifested by the increase of the bond distance and the decrease of the H-H Raman active vibrational frequency are well characterized for the first time.

We thank the referee for acknowledging the interest of the study.

The identification of some of the Raman modes to "libration" needs to be further explained and put into context. These are presumably k=0 phonon modes. How many such modes are there and what are their symmetries? The librational motion and its relationship to rotation in high pressure H2 has been extensively studied. However, in the presented crystal structures the motions of the H2 molecules relative to the heavy ions/atoms needs further clarification. Just to say "libration" does not do full justice to what the nature of these modes are, although Fig S11 is helpful, but there are more phonon modes than shown there.

We refer to modes associated with the angular degrees of freedom of the molecules as "libration". As shown by Cooke et al. Comp. Mat. Sci.. 2022; 210,111400). the quantum wavefunctions associated with these modes can be interpreted as vibrations, rotations or reorientations. In the present work, the precise nature of the mode is of secondary importance as we use this as additional evidence for changes in the crystal structure/composition

The article mentions that "The most intense band, above 3000 cm-1 (Fig. 3), corresponds to the A1g mode resulting from the H-H intramolecular stretching of the H2, vibron (see Fig. 3 and Fig. S11). How was the symmetry assigned? (See also Table S2.) The references given in this context are not shedding light on the symmetry assignments of these modes. [19, 33, 38, 39] Can you provide the information about the symmetry assignment?

The symmetry is assigned - they are computed by Castep as part of the Raman calculation, as implemented by Keith Refson (Com. Phys. Comm. 1972). We included this information and reference in the text. The visualization of the modes within the lattice was done using Jmol.

Minor points:

Can you provide figures for the relative intensities of the Raman modes as a function of pressure?

Analysis of the intensities provides information when comparing to neighbouring modes. Here we do not see any intensity redistribution between modes upon compression. This is why the intensity analysis is not provided, and feel it would add confusion to the main message we want to deliver.

Figure 1a: Two slightly different values of the wavelength are given. Please correct.

The results are taken from two different experiments which did have differing wavelengths.

Figure 1b: The DFT values are represented by four dashed lines. This is very nice and helpful. However, the computations provide only discreet points on these curves. Similar problems arise in Fig. S1a and others.

We changed the theoretical values to symbols.

Please provide in the SI section a table listing the specific values (pressures and the respective volumes in order to document the DFT computations more completely.

We have added this and can be viewed in table S4.

The two curves for "DFT, I4/mmm, BaH4", and "DFT R3m BaH4" seem to overlap. Use symbols that help the reader to see if this is really the case, e.g. dashed line for one, and empty triangles for the other.

We agree with the referee, thanks for pointing this out. We have now plotted I4/mmm BaH4 as hollow squares and the R3m BaH4 as asterisks.

Could the line for "DFT, I4/mmm , CaH4" be continued up to 70 GPa, since the experimental data extend that far?

We have added volumes theoretically calculated for CaH_4 up to 75 GPa.

I recommend to avoid using the term "hydrogen bond" in the title. This is article is about bonding in dihydrogen.

We have changed this to intramolecular hydrogen bond or covalent bond where possible.

Reviewer: 2

Comments:

The effect of chemical and hydrostatic pressure has been the subject of intense collateral research in the analysis of hydrogen superconductors. This paper aims at providing direct insight into the issue from the analysis of the Ca-Sr-Ba family (same crystalline structure). It is thus a timely and necessary investement that was missing in the literature. Hence, I suggest its publication under minor revisions:

We thank the referee for the positive review and valuing the insights our manuscripts provide.

-The sentence "electronic weakening of the bond by filling the antibonding orbital, sigma*, and physical weakening by conning the molecule to small interstitial locations" is slightly missformulated. Both the electronic effects (charge transfer) and confinement lead to filling of the antibonding orbital.

Thank you very much for pointing this out. We have changed it: electronic effects (charge transfer) and confining the molecule to small interstitial locations, so-called chemical pre-compression.

-More details should be given on how the authors measured the ELF radius. It would also be interesting to measure the volume of the octahedral and tetrahedral cages instead when commenting on their sizes.

We have included a sentence in the SI "We have also calculated ....alkaline-earth metal" to explain how the ELF radius is calculated. We have also added table S5 with the values for the volumes of the basins associated to the octahedral and tetrahedral ELF maxima of Fig. 2.

-Differences in distances and c/a ratio between experiment and theory are found. Did the authors try other functionals or including dispersion? This should be commented on the method section.

In our calculations we have used the PBE functional to facilitate comparison with previous works where it has been extensively used. We have also tested the BLYP functional and considered van der Waals contributions with PBE-TS. These functionals give slightly different volumes, c/a ratios and

frequencies, the differences being a typical of the accuracy of DFT. Result for alternative functionals have been included in the SI.
